# SLC1A5 Prefers to Play as an Accomplice Rather Than an Opponent in Pancreatic Adenocarcinoma

**DOI:** 10.3389/fcell.2022.800925

**Published:** 2022-03-28

**Authors:** Fangshi Xu, Hai Wang, Honghong Pei, Zhengliang Zhang, Liangliang Liu, Long Tang, Shuang Wang, Bin-Cheng Ren

**Affiliations:** ^1^ Department of Medicine, Xi’an Jiaotong University, Xi’an, China; ^2^ Department of Emergency, The Second Affiliated Hospital of Xi’an Jiaotong University, Xi’an, China; ^3^ Department of Emergency, Shaanxi Provincial People’s Hospital, Xi’an, China; ^4^ Department of Dermatology, The Second Affiliated Hospital of Xi’an Jiaotong University, Xi’an, China; ^5^ Department of Rheumatology and Immunology, The Second Affiliated Hospital of Xi’an Jiaotong University, Xi’an, China

**Keywords:** pancreatic adenocarcinoma, ferroptosis, SLC1A5, glutamine, mTORC1, prognosis, tumor immune microenvironment

## Abstract

**Background:** SLC1A5, a ferroptosis regulator gene, plays a dual role in cancer regulation. However, the roles of SLC1A5 in pancreatic adenocarcinoma (PAAD) remain elusive.

**Methods:** SLC1A5’s expression and somatic mutation information were determined by TCGA, GEO, Oncomine, and cBioPortal databases. Its prognostic value was assessed in TCGA cohort and was validated in three independent cohorts. The effects of SLC1A5 on the tumor immune microenvironment were analyzed by the CIBERSORT algorithm, ssGSEA method, and TISIDB and TIMER databases. The “oncoPredict” R package, TIDE algorithm, ImmuCellAI online tool, and GSE35141 and GSE59357 datasets were used to ascertain its therapeutic correlations. GSEA and Western blot were applied to reveal the effects of SLC1A5 on the mTORC1 signaling pathway and ferroptosis process. The biofunctions of SLC1A5 were assessed by MTT, wound-healing, Transwell, and xenograft assays.

**Results:** SLC1A5 was significantly upregulated in the PAAD samples but was not commonly accompanied with somatic mutation (2.3%). Overexpression of SLC1A5 led to a poor prognosis and was identified as an independent prognostic factor. Moreover, high SLC1A5 expression suppressed the antitumor immune process by changing the infiltrating levels of immune cells. As for therapeutic correlations, SLC1A5 was related to the efficacy of dasatinib, sunitinib, sorafenib, and imatinib but may not predict that of radiotherapy, chemotherapeutic drugs, and immune checkpoints inhibitors (ICIs). Notably, the overexpression of SLC1A5 could activate the mTORC1 signaling pathway and may increase the cellular sensitivity to ferroptosis. Finally, the overexpression of SLC1A5 markedly promoted proliferation, migration, and invasion of pancreatic cancer cells. At the *in vivo* level, SLC1A5 deletion inhibited tumor growth in a mice xenograft model.

**Conclusions:** SLC1A5 prefers to play as an accomplice rather than an opponent in PAAD. Our findings provide novel insights into PAAD treatment.

## 1 Introduction

Pancreatic adenocarcinoma (PAAD) is a highly malignant abdominal cancer and is the seventh leading cause of cancer-related death around the world (Bray et al., 2018). To date, surgery has been the most effective approach for treating PAAD. However, more than half of the cases already present metastatic signs at the time of diagnosis, which leads these patients to be unresectable or not suitable for surgery (Balsano et al., 2019). Although substantial efforts have been made for improving patient prognosis, the 5-year overall survival rate (OSR) of PAAD patients is commonly less than 9% (Huang et al., 2021). The first-line chemotherapy options, such as FOLFIRINOX, gemcitabine, and nab-paclitaxel, do not completely fulfill the urgent expectations for cures. The median OS of metastatic pancreatic cancer (mPC) patients receiving FOLFIRINOX or gemcitabine plus nab-paclitaxel treatments were only 12.7 and 10.2 months, respectively (Franco et al., 2021). Unfortunately, the efficacy of PD-1/PD-L1 (programmed cell death protein/ligand) blockade treatment is also dismal. The objective response rate of the treated patients ranges only from 6% to 17% (Brahmer et al., 2012). Recently, numerous lines of research evidence have witnessed the crucial roles of ferroptosis in the cancer onset and progression, which has a great potential to remedy the therapeutic limitations of current means (Su et al., 2020).

Ferroptosis, a novel mode of programmed cell death, heralds a new dawn for cancer treatment (Hassannia et al., 2019). As acknowledged, ferroptosis is driven by three crucial links, namely, iron dependence, antioxidant imbalance, and lipid peroxidation, all of which are regulated by multiple genes (Cao and Dixon 2016; Xie et al., 2016). Therefore, these genes were also suggested to participate in the proliferation and apoptosis of tumor cells. For example, NCOA4, a ferritinophagy regulatory gene, leads to unfavorable prognosis and defective immune cell infiltration in renal cancer (Mou et al., 2021). SLC7A11, a cystine/glutamate antiporter, not only enhances cervical cancer progression by circEPSTI1-miR-375-SLC7A11 axis (Wu et al., 2021) but also serves as an unfavorable prognostic biomarker in renal carcinoma (Xu et al., 2021a). Alternatively, SLC1A5 has been identified as a positive modulator of ferroptosis (Cao and Dixon 2016). It is now well established that SLC1A5 can increase glutamine uptake, thus facilitating a-ketoglutarate generation (Gao et al., 2015). Next, the accumulation of a-ketoglutarate can contribute to the oxidization of membrane lipids by promoting the synthesis of the corresponding fatty acid precursor (Dixon et al., 2012). As a result, SLC1A5 is capable of inhibiting tumor growth by supporting ferroptosis (Lin et al., 2018; Luo et al., 2018).

However, the regulatory roles of SLC1A5 in cancer are not straightforward; it is a double-edged sword in cancer progression (Figure 9D). Besides acting as a positive regulatory gene of ferroptosis against cancer, another identity of SLC1A5 is that it is a glutamine transporter. Glutamine is a nonessential amino acid, but it is essential for tumor growth. Elevated glutamine consumption is one of most critical metabolic hallmarks of a tumor (Matés et al., 2019). Since SLC1A5 is responsible for intracellular glutamine supply, it is pivotal for maintaining the energetic demands from vigorous cancer cells (Liu et al., 2018). It is now well established that SLC1A5 is upregulated and promotes cancer development in multiple cancers, such as hepatocellular carcinoma (HCC), non-small-cell lung cancer (NSCLC), and gastric cancer (GC) (Lopes et al., 2021). Taking these findings together, SLC1A5 has a dual role in cancer regulation. So, which camp does SLC1A5 exactly belong to in PAAD? In the current study, we comprehensively analyzed SLC1A5’s expression, prognostic value, immune effect, therapeutic correlation, and biofunctions in PAAD through bioinformatic and experimental means. SLC1A5 was not only a biomarker for unfavorable prognosis, it also could suppress the antitumor immune process. More importantly, we found that SLC1A5 could enhance proliferative, migrative, and invasive abilities of pancreatic cancer (PC) cells, while it may increase the sensitivity to ferroptosis. Our findings ascertained that SLC1A5 served as an accomplice in PAAD.

## 2 Materials and Methods

### 2.1 Data Source

Multiple public databases, including TCGA (https://portal.gdc.cancer.gov/), ICGC (https://dcc.icgc.org/releases), and GEO (https://www.ncbi.nlm.nih.gov/geo/), provide clinical information and transcriptome data for bioinformatic analyses. The TCGA dataset (discovering cohort) consists of 178 PAAD and four normal samples. The workflow type of transcriptome profiling and the data format of clinical information are “HTSeq-FPKM” and “bcr xml,” respectively. Due to the insufficient normal samples in the TCGA database (*n* = 4), we supplemented 167 normal pancreatic samples from the GTEx database (https://xenabrowser.net/datapages/). GSE71729, GSE62165, GSE16515, GSE43795, GSE62452, GSE21501, GSE35141, and GSE59357 datasets were applied for gene expression, prognostic validation, and therapeutic response analyses. The specific uses, descriptions, and sample sizes of these GEO datasets are shown in Table 1. The PACA-AU project in the ICGC database was selected to serve as a validation cohort which contained 81 available PAAD samples (the samples have both transcriptomic data and clinical information). To ensure comparability between different datasets, the gene expression data were standardized by log2 transformation.

**TABLE 1:** The uses of datasets in the present study.

Dataset	Sample size T/N	PMID	Uses
TCGA	178/4	—	Training cohort for analyzing the expression, prognostic value, and immune effect of SLC1A5
GSE71729	145/46	26343385	Expression analysis
GSE62165	118/13	27520560	Expression analysis
GSE16515	36/16	27749787	Expression analysis
GSE43795	26/5	24072181	Expressive information of SLC1A5 in three PC subtypes
GSE62452	69/61	27197190	1.Expression analysis
			2.Validation cohort for evaluating the prognostic value of SLC1A5
ICGC-AU project	81/0	—	Validation cohort for evaluating the prognostic value of SLC1A5
GSE21501	102/0	20644708	Validation cohort for evaluating the prognostic value of SLC1A5
GSE35141	GEM-resistant and parental PC cells	2490663	Predicting the therapeutic efficacy
GSE59357	Dasatinib-resistant and -sensitive PC cells	25637283	Predicting the therapeutic efficacy

T/N, tumor samples versus normal samples; PC, pancreatic cancer; GEM, gemcitabine.

### 2.2 Selecting SLC1A5 as the Study Subject

We progressively reduced the scope of the study candidates through a four-step process. First, a ferroptosis-related (FR) gene set comprising 64 FR genes was constructed based on some critical reviews (Xie et al., 2016; Mou et al., 2019; Hassannia et al., 2019; Li et al., 2020; Cao and Dixon 2016) and previous studies (Wu et al., 2020; Hong et al., 2021; Jiang et al., 2021; Xu et al., 2021b). The STRING database (https://string-db.org/) and Cytoscape software (ver. 3.4.0) (Doncheva et al., 2019) were utilized to construct the protein–protein interaction (PPI) network of these FR genes. Then, the most significant module in the PPI network was screened out by Cytoscape plugin MCODE (Rivera et al., 2010). Second, FR differential expressed genes (DEGs) (absolute value of Log_2_FC ≥ 0.5) were identified by the “Limma” package in R software (ver. 3.6.3). Third, the cox univariate regression analysis was performed to find out the PAAD prognostic genes. Finally, the intersection among the hub module, DEGs, and prognostic genes was obtained by a Venn diagram. Given that SLC1A5 has attracted prominent concerns in oncology in recent years (Liu et al., 2018), we selected SLC1A5 as the primary research goal.

### 2.3 Expression and Mutation Analyses

Regarding the high heterogeneity of patients’ samples, the expressive difference of SLC1A5 between PAAD and normal samples was determined by the Wilcoxon rank-sum test. Using the Oncomine database (https://www.oncomine.org/), we assessed SLC1A5 expressions in pan-cancers and conducted a meta-analysis to reconfirm the SLC1A5 expressive trend based on two PAAD datasets. The analytical thresholds are as follows: *p*-value = 0.05, fold change = 1.5, gene rank was “Top 20%,” and data type was “mRNA.” In addition, the somatic mutation information of SLC1A5, including the mutation frequency and types, was obtained from the cBioPortal database (http://cbioportal.org) (Cerami et al., 2012).

### 2.4 Prognostic Analyses

The optimal cutoff value of SLC1A5 expression was calculated by the Cutoff Finder online tool (http://molpath.charite.de/cutoff) (Budczies et al., 2012). PAAD samples in different cohorts were all divided into high- and low-SLC1A5-expression groups according to the cutoff value. Survival difference analyses were conducted based on the Kaplan-Meier method. The receiver operating characteristic curve (ROC) was applied to evaluate the predictive performance of SLC1A5 and other clinical parameters. The decision curve analysis (DCA) was used to assess the clinical gain effect brought by SLC1A5 on the traditional prognostic model of PAAD. The traditional prognostic model was composed of age, gender, histological grade, and clinical stage based on the multivariate cox regression algorithm. Through univariate and multivariate cox analyses, we identified the independent prognostic factors of PAAD. Clinical subgroup analyses were performed to estimate the applicable range of SLC1A5 in the PAAD prognostic analysis. Moreover, we constructed a nomogram combining PAAD clinicopathological features (age, histological grade, and TN staging) and the SLC1A5 expressive level to predict the overall survival rate (OSR) of individual at 1, 3, and 5 years. A calibration plot was employed to compare the predicted and actual survival probabilities. As the number of PAAD patients with M0 and clinical III-IV stages in the TCGA cohort was only two and seven, respectively, we did not perform subgroup analyses for these cases.

The prognostic value of SLC1A5 was also tested in three validation cohorts, namely, GSE62452, GSE21501, and ICGC-AU cohorts. Survival difference analysis and ROC were performed in each cohort. The grouping criteria of the PAAD patients were consistent with the TCGA ones.

### 2.5 Immune Analyses

As for the TCGA cohort, the abundance of 22 immune cells in each PAAD sample was obtained using the CIBERSORT algorithm (Newman et al., 2019). Then, the differences in the cellular infiltration levels between different SLC1A5 expression groups were determined via the “Limma” package in R software. Based on the ssGSEA (single-sample gene set enrichment analysis) method, the activities of eight immune-related pathways were quantified by the “GSVA” package (Hänzelmann et al., 2013). The distributions of SLC1A5 expressions across different immune subtypes were exhibited via the “Subtype” tab in the TISIDB database (http://cis.hku.hk/TISIDB/) (Ru et al., 2019). The correlation between the enrichment level of antitumoral immune effector cells and SLC1A5 expression were uncovered by the Spearman correlation test. The TIMER web tool (https://cistrome.shinyapps.io/timer/) (Li T. et al., 2017) was implemented to determine the relationship between the somatic copy number alteration (SCNA) of SLC1A5 and infiltration levels of six crucial immune cells.

Furthermore, the immune effects of SLC1A5 were also assessed in three external cohorts (GSE62452, ICGC-AU, and GSE21501). The differences in the enrichment of 22 immune cells in the external cohorts were ascertained through the “ImmuCellAI” online tool (http://bioinfo.life.hust.edu.cn/ImmuCellAI#!/) (Miao et al., 2020).

### 2.6 Therapeutic Response Analyses

In this study, we investigated the predictive effects of SLC1A5 on the efficacy of multiple therapeutic approaches. Using the clinical information from the TCGA cohort, expressive difference of SLC1A5 between the radiotherapy-response and -non-response patients was analyzed by the Wilcoxon test. GSE35141 and GSE59357 provided gene expression profiles of sensitive and resistant cell lines to gemcitabine (GEM) and dasatinib, respectively. The associations between the SLC1A5 expression and different drug responses were also explored by the Wilcoxon test. oncoPredict is an R package for predicting the drug response (Maeser et al., 2021), by which the associations of SLC1A5 expression with the sensitivities to the commonly utilized chemotherapy and molecular targeted drugs were investigated.

As for immune checkpoint inhibitors (ICIs), considering that patients with overexpression of immune checkpoints (ICs) have a greater propensity for benefiting from ICI therapy (Yuasa et al., 2017), we analyzed the expressive correlations between SLC1A5 and six pivotal ICs (PD-1, CTLA4, LAG3, HAVCR2, TIGIT, and BTLA) based on the Spearman test. Moreover, Jiang P’s team has developed the novel signatures of T-cell dysfunction and exclusion to predict the cancer immunotherapy response (Jiang et al., 2018) (http://tide.dfci.harvard.edu/). The tumor immune dysfunction and exclusion (TIDE) score of each PAAD sample in the TCGA cohort was calculated and the differences in the TIDE score between high and low SLC1A5 expressions were determined. Finally, ImmuCellAI, a method that has the ability to predict the response of ICI therapy through analyzing the distribution and abundance of immune cells, especially T-cell subsets, was also applied to clarify the potential linkages between SLC1A5 and the therapeutic response of ICIs (Miao et al., 2020) (http://bioinfo.life.hust.edu.cn/ImmuCellAI#!/).

### 2.7 GSEA

GSEA (gene set enrichment analysis) was utilized to unravel the impacts of SLC1A5 on the ferroptosis process, glutamine metabolism, and mTORC1 signaling pathways. The analytical gene sets were obtained from the Molecular Signatures Database (MSigDB) (Liberzon et al., 2015), including “WP Ferroptosis,” “Hallmark Oxidative Phosphorylation,” “GOBP Glutamine Family Amino Acids Biosynthetic Process,” and “Hallmark mTORC1 signaling.” The phenotype labels were set as high-expression SLC1A5 samples versus low-expression ones. The number of permutations was set as 1,000. There was no collapse in gene symbols. The descriptions of these gene sets are shown in Table 2.

**TABLE 2:** Description of the gene sets for GSEA.

Names	Gene counts	Description
Hallmark mTORC1 signaling	200	Genes upregulated through activation of the mTORC1 complex
GOBP Glutamine Family Amino Acids Biosynthetic Process	17	The chemical reactions and the pathways resulting in the formation of amino acids of the glutamine family, comprising arginine, glutamate, glutamine, and proline
WP-ferroptosis	40	Genes responsible for the ferroptosis regulation
Hallmark oxidative phosphorylation	200	Genes encoding proteins involved in the Oxidative Phosphorylation

GSEA, gene set enrichment analysis; GO, gene ontology; BP, biological process; WP, WikiPathways.

### 2.8 Cell Culture and Transfection

Two human pancreatic cancer cell lines (PANC-1 and SW1990) and a normal pancreatic duct epithelia cell line (HPDE6-C7) were purchased from Procell Life Science&Technology (Wuhan, China). PANC-1 cells were cultured in DMEM (Dulbecco’s Modified Eagle Medium) containing 10% FBS (fetal bovine serum) and 1% P/S (penicillin/streptomycin) (Procell, Wuhan, China). SW1990 cells were cultured in Leibovitz’s L-15 medium containing 10% FBS and 1% P/S (Procell, Wuhan, China). HPDE6-C7 cells were cultured in DMEM (Dulbecco’s Modified Eagle Medium) containing 10% FBS and 1% P/S (Procell, Wuhan, China). The culture conditions were 37°C with 5% CO_2_.

SLC1A5-specific shRNA and amplification plasmids were designed by Hanheng Biotechnology (Shanghai, China). Lentiviruses were also purchased from Hanheng Biotechnology (Shanghai, China) and were applied to transfect cells. The interference efficiency was tested by RT-qPCR after 72 h post-transfection.

### 2.9 Real-Time Quantitative PCR

Total RNA was extracted using TRIzol-Chloroform method (Invitrogen, United States). The RNA concentration was measured by A260/A280 ratio (Nanodrop 2000 spectrophotometer). Inverse transcription was performed by PrimeScript RT Reagent Kit (TaKaRa, Japan). RT-qPCR was tracked by SYBR-Green PCR Reagent (Takara, Japan) and performed on the ABI Prism 7900 sequence detection system. GAPDH was selected as an internal reference. The RNA relative expression was calculated based on the 2^−ΔΔCT^ method. The primer lists and the Ensembl number of SLC1A5 were presented in Supplementary Table S1.

### 2.10 MTT Assay

After trypsin digestion, the log-phase growing cells were made into single-cell suspension by a medium containing 10% FBS. 5 × 10^3^/per well cells were seeded into 96-well plates. The MTT assay was performed to detect cell proliferation at time points of 24, 48, 72, and 96 h, respectively. At each time point, the MTT Reagent (Solarbio, Beijing, China) was added to each well and incubated at 37°C for 4 h. The supernatant was removed and 150 μl DMSO was added for dissolving formazan crystals. The absorbance was measured at 490 nm.

### 2.11 Wound-Healing Assay

The transfected cells were seeded into six-well plates (5 × 10^4^ cells per well). When they reached more than 90% confluence, a linear wound was created by a sterile pipette tip. The cells were incubated in a serum-free medium for 24 h. After washing twice with PBS, the migration process was observed by a microscope. The migratory abilities of the cells were quantified by the wound width rate. Relative migration = (the scratch width at 24 h *divided by* that at 0 h) × 100%.

### 2.12 Transwell Assay

In this assay, 24-well Transwell chambers (Corning, NY, United States) precoated with Matrigel were applied to assess the cell invasive ability. A serum-free medium and 10% FBS medium were added to the upper and lower chambers, respectively. The transfected cells (1 × 10^5^ cells per well) were placed in the upper chamber and incubated for 24 h. After discarding the medium in the upper chamber, the non-invasive cells were removed by washing twice in PBS and wiping with cotton swab. The invasive cells attached to the lower side of the membrane were fixed with 4% paraformaldehyde for 15 min. Then, they were stained by 0.1% crystal violet for 5 min at room temperature. The stained cells were counted at 100-fold magnification per five random fields of view under a microscope.

### 2.13 Western Blot

After twice PBS washing, the transfected cells were lysed on ice by RIPA buffer (Beyotime, China). Centrifugation was performed at 12,000 rpm for 4 min. Then, the supernatant was collected and moved into an EP tube. The protein concentration was measured by a BCA kit (Phygene Life Sciences Company, Fuzhou, China). The sample absorbance at 562 nm was measured after 30-min incubation with a BCA working reagent. The protein concentration of each sample was calculated according to the standard curve. The sample proteins were separated by 10% SDS-PAGE. After electrophoresis, the protein samples were transferred to PVDF membranes (BestBio, Shanghai, China). The PVDF membranes were blocked by 5% skimmed milk at 37°C for 2 h and then were washed by the TBST buffer (BIOSIC, Nanjing, China) for three times. The treated membranes were incubated with the primary antibody overnight at 4°C. After three times TBST washing, the membranes were incubated with the secondary antibody. The primary and secondary antibodies were all purchased from Abcam (Shanghai, China). The primary antibodies were as follows: rabbit monoclonal anti-SLC1A5 (1:2000, ab237704), anti-CDK4 (1:1000, ab199728), anti-TFRC (1:1000, ab214039), anti-mTOR (1:1000, ab134903), anti-p-mTOR (1:1000, ab109268), anti-4EBP1 (1:1000, ab32024), anti-p-4EBP1 (1:1000, ab75767), anti-p70S6K (1:1000, ab186753), anti-p-p70S6K (1:500, ab59208), and rabbit polyclonal anti-GAPDH antibody (1:1000, ab22555). The secondary antibody was goat-anti rabbit IgG-HRP secondary antibody (1:2000, ab205718).

### 2.14 Xenograft Assay

Six-week-old female BALB/c nude mice were applied to conduct tumor xenograft experiments. PANC-1 cells with stably expressing SLC1A5 and sh-SLC1A5 were injected subcutaneously into the flanks of each mouse. The injection concentration and volume were 5 × 10^7^ cells/ml and 100 μl, respectively. The tumor volume was calculated as 0.5 × (tumor length) × (tumor width)^2^. The tumor length and width were measured by a vernier caliper every 7 days. After 5 weeks, all mice were euthanized, and xenograft tumors were collected. Meanwhile, the protein expressions of SLC1A5 and some ferroptosis markers (including TFRC and SLC7A11) in xenograft tumors were evaluated by Western blot. The experimental approach was analogous to that described in *Western Blot*. Other required antibody was as follow: rabbit monoclonal anti-SLC7A11 (1:1000, ab175186). This study was approved by the Animal Ethics Committee of the Second Affiliated Hospital of Xi’an Jiaotong University.

## 3 Results

**FIGURE 1:**
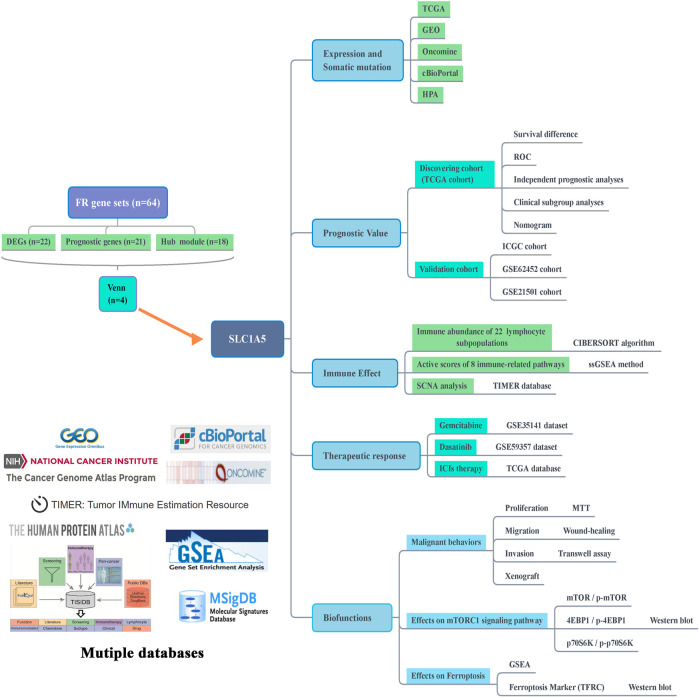
Flow chart of this study. FR, ferroptosis-related; DEGs, differentially expressed genes; TCGA, The Cancer Genome Atlas; GEO, Gene Expression Omnibus; HPA, Human Protein Atlas; ROC, receiver operating characteristic curve; ICGC, International Cancer Genome Consortium; ssGSEA, single-sample gene set enrichment analysis; SCNA, somatic copy number alteration; ICIs, immune checkpoint inhibitors; GSEA, gene set enrichment analysis; TFRC, transferrin receptor.

In this study, we firstly screened out SLC1A5 as the pivotal regulatory gene in the ferroptosis process through expression, prognosis, and module analyses. Subsequently, we comprehensively probed into SLC1A5 functions in PAAD from the genetical expression, prognostic value, immune effect, and curative effect prediction perspectives. More importantly, its malignant biofunctions and associations with the mTORC1 signaling pathway and ferroptosis were ascertained by *in vitro* and *in vivo* experiments. The flow chart is shown in Figure 1. The clinical characteristics of TCGA, ICGC, and GEO cohorts are presented in Supplementary Tables 2–5.

### 3.1 SLC1A5 Plays a Central Role in the Ferroptosis Regulatory Network

**FIGURE 2:**
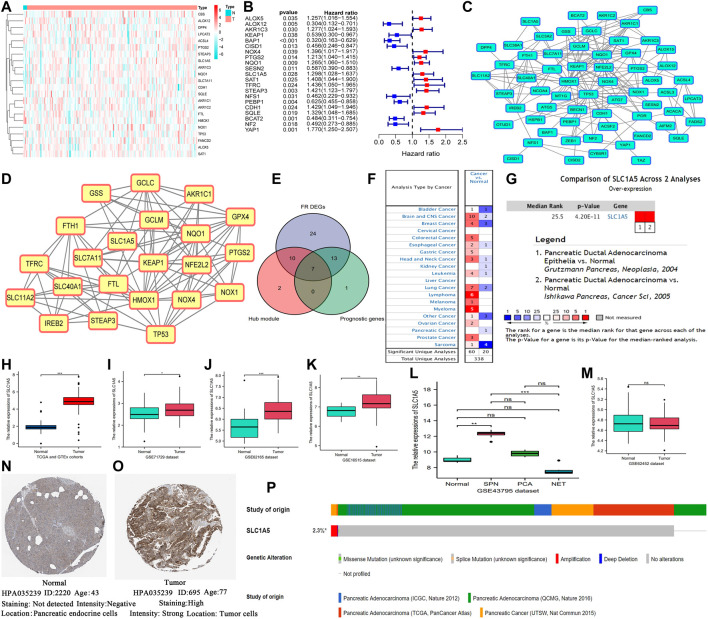
SLC1A5 is identified as a crucial regulator in ferroptosis and is significantly upregulated in PAAD. **(A)** The heatmap of ferroptosis DEGs. **(B)** Prognosis-related ferroptosis genes. **(C)** The PPI network of 64 FR genes. **(D)** The most significant module in ferroptosis PPI network. **(E)** The intersection among the hub module, DEGs, and prognostic genes. **(F)** Pan-cancer analysis of SLC1A5 based on the Oncomine database. **(G)** A meta-analysis of SLC1A5 expression based on two pancreatic cancer datasets. **(H)** The expressive difference of SLC1A5 between the normal and PAAD samples in the TCGA cohort. **(I–M)** The expressive differences of SLC1A5 between the normal and PAAD samples in multiple GEO datasets. **(N,O)** The histological expression of SLC1A5 based on the HPA database. **(P)** The somatic mutation information of SLC1A5 based on the cBioPortal database. PAAD, pancreatic adenocarcinoma; DEGs, differentially expressed genes; PPI, protein-protein interaction; FR, ferroptosis-related; NS, no statistical differences; *p < 0.05, **p < 0.01, and ***p < 0.001.

There were significant differences in the expression of most of the ferroptosis regulatory genes (FRGs) (84.4%, 54/64) between the normal and PAAD samples (Figure 2A). Among them, only CBS was markedly downregulated (Log_2_FC = –6.324), while 44 other FRGs were upregulated (SLC1A5, Log_2_FC = 3.124). Besides, 21 of 64 FRGs profoundly affected the prognosis of PAAD patients (Figure 2B). In total, 12 FRGs were unfavorable for survival outcomes (SLC1A5, HR = 1.298), whereas the rest served as protective prognostic factors. The PPI network of the ferroptosis regulators is displayed in Figure 2C. Next, we selected the core functional subnet module which consisted of 19 nodes and 101 edges from the PPI network (Figure 2D). Finally, the intersection of DEGs, prognostic genes, and hub module genes was generated, including KEAP1, NOX4, TFRC, NQO1, PTGS2, STEAP3, and SLC1A5 (Figure 2E). Since SLC1A5 has abilities to transport glutamine required for cell proliferation and to inhibit ferroptosis by promoting a-ketoglutarate synthesis (Cao and Dixon 2016; Jiang et al., 2020), it has become a hotspot in tumor research in recent years. Therefore, we focused on its functions in PAAD.

### 3.2 SLC1A5 Is Significantly Overexpressed in Pancreatic Carcinoma

The Oncomine pan-cancer analysis revealed that SLC1A5 overexpression widely occurred in multiple cancers (Figure 2F). The meta-analysis of two PAAD datasets (Grützmann et al., 2003; Ishikawa et al., 2005) also confirmed its overexpression in PAAD samples (Figure 2G). Moreover, the same expressive alterations were observed in both the TCGA and GEO datasets (Figures 2H–K). The Wilcoxon rank-sum test results of each dataset are shown in Supplementary Table 6. Especially in the GSE43795 dataset, SLC1A5 was upregulated in two subtypes of PC, namely, solid-pseudopapillary neoplasm of pancreas (SPN) and pancreatic ductal adenocarcinoma (PCA), but was downregulated in the neuroendocrine tumor (NET) (Figure 2L). Interestingly, there was no significant difference in SLC1A5 expression between the normal and PAAD samples in the GSE62452 dataset (Figure 2M). As for protein expression, SLC1A5 was strong staining in tumor tissues but negative staining in normal tissues (Figures 2N,O). Besides, the somatic mutation of SLC1A5 was not common in the PAAD samples (2.3%), and amplification was the most predominant mutational type (Figure 2P).

### 3.3 SLC1A5 Is a Critical Complement to the PAAD Prognostic Assessment

**FIGURE 3:**
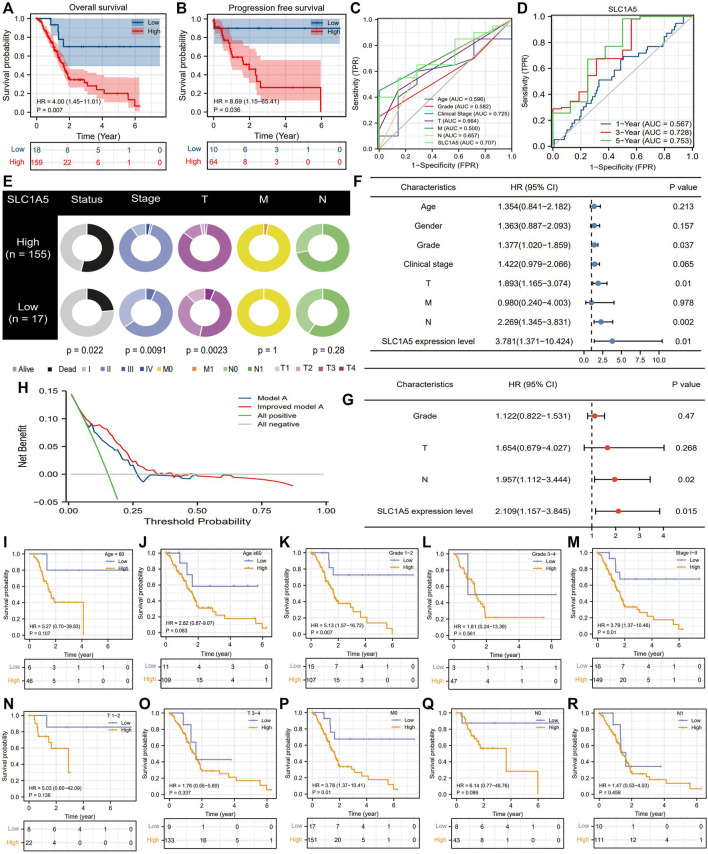
SLC1A5 possesses a great prognostic value. **(A)** OS difference between high- and low-SLC1A5-expression groups. (B) PFS difference. **(C)** ROCs. **(D)** Time-independent ROCs. **(E)** The relationship between SLC1A5 expression and clinicopathological features of PAAD. **(F,G)** Independent prognostic analyses based on the multivariate and univariate cox regression methods. **(H)** DCA results. “Model A” represents the traditional prognostic model consisting of age, gender, histological grade, and clinical stage (blue line). “Improved model A” represents the prognostic model consisting of age, gender, histological grade, clinical stage, and SLC7A11 expression (red line). **(I–S)** Clinical subgroup analyses. OS, overall survival; PFS, progression-free survival; ROC, receiver operating characteristic curve; PAAD, pancreatic adenocarcinoma; DCA, decision curve analysis.

According to the optimal cutoff value of SLC1A5 relative expression (4.039), 177 PAAD patients from the TCGA cohort were divided into high- and low-SLC1A5-expression groups (Supplementary Figure 1). High SLC1A5 expression led to unfavorable survival outcomes, including overall survival (OS) and progression-free survival (PFS) (Figures 3A,B). ROC indicated that SLC1A5 possessed an excellent predictive performance which had a certain preponderance over conventional clinicopathological features, such as age, histopathological grade, and TNM-staging (AUC = 0.707) (Figure 3C). Time-dependent ROC revealed that the predictive accuracy of SLC1A5 increased with time, reaching 0.753 for the 5-year OS (Figure 3D). Besides, high SLC1A5 expression was positively correlated with more advanced clinical and T stages (Figure 3E). Through univariate and multivariate cox analyses, SLC1A5 (HR = 2.109, *p* = 0.015) and N stage (HR = 1.957, *p* = 0.02) were identified as the independent prognostic factors of PAAD (Figures 3F,G).

**FIGURE 4:**
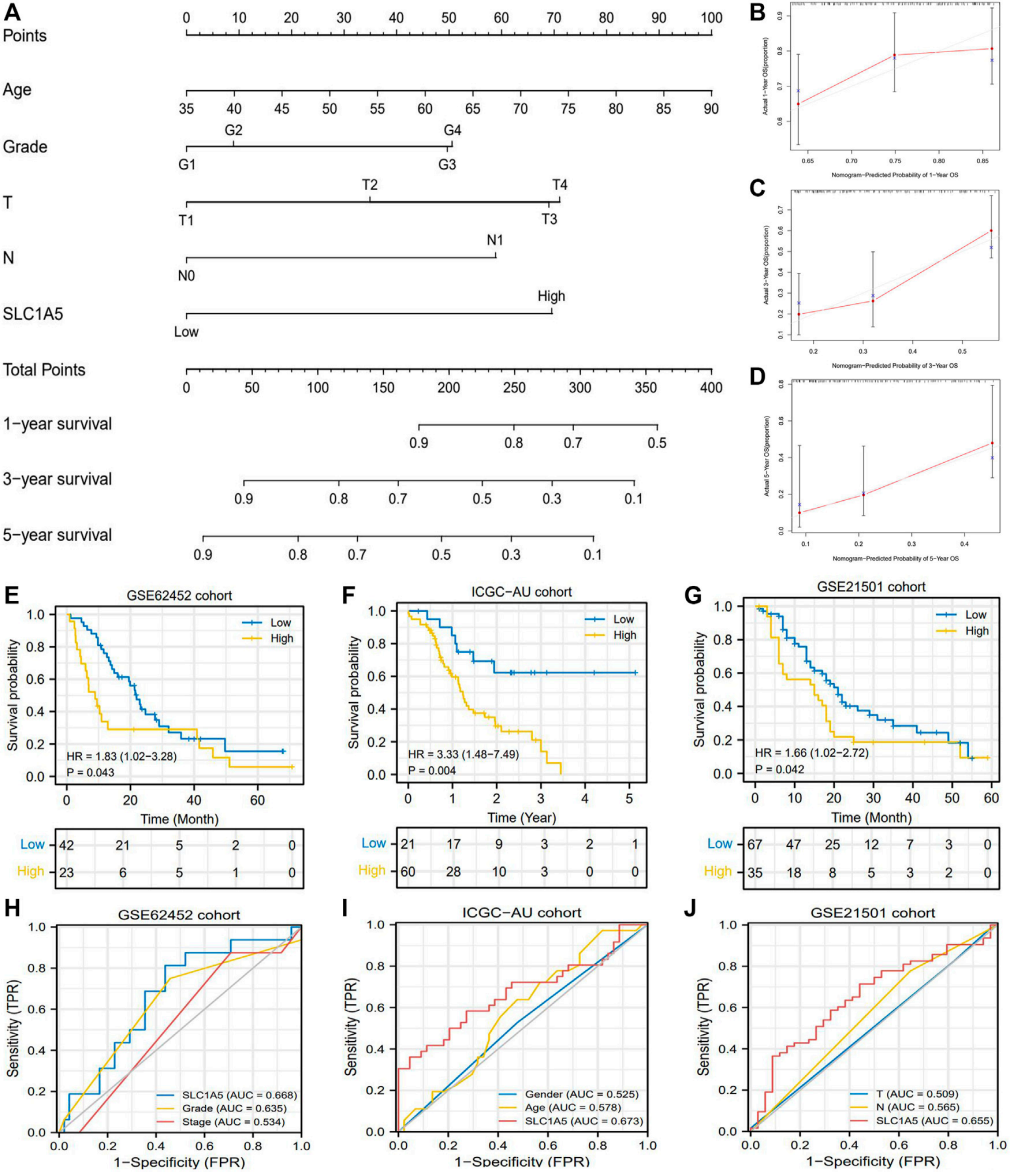
The prognostic value of SLC1A5 is validated in multiple cohorts. **(A)** The nomogram predicting the 1-, 3-, and 5-year overall survival probability of PAAD patients. **(B–D)** Calibration curve of the nomogram. **(E)** OS difference between high- and low-SLC1A5-expression groups in the GSE62452 cohort. **(F)** OS difference in the ICGC-AU cohort. **(G)** OS difference in the GSE21501 cohort. **(H)** ROCs of the GSE62452 cohort. **(I)** ROCs of the ICGC-AU cohort. **(J)** ROCs of the GSE21501 cohort. PAAD, pancreatic adenocarcinoma; OS, overall survival; ROC, receiver operating characteristic curve; FPR, false positive rate.

Introduction SLC1A5 expression into the traditional prognostic model could increase the benefit of clinical decision-making to some extent (Figure 3H). The C-index of the improved model was 0.727; in contrast, that of the traditional model was 0.669 (Supplementary Table 7). Regarding the prediction range, SLC1A5 was able to distinguish the prognostic differences of patients with histopathological grades 1–2, clinical stage I-II, and MO stages but failed in other clinical subgroups (Figures 3I–S). To provide a convenient and straightforward access to predict the 1-, 3-, and 5-year overall survival rates (OSR) of PAAD patients, we constructed a nomogram consisting of age, histopathological grade, TN stages, and SLC1A5 expression (Figure 4A). For example, 60-year-old PAAD patients who are diagnosed as G2, T2, and N0 stages, with high SLC1A5 expression, will get about 165 points in total, which corresponds to a 3-year OSR of 70%. Meanwhile, the calibration plots showed that the predicted probabilities well matched with the actual survival rate, especially for the 5-year OSR (Figures 4B–D). In light of the above, SLC1A5 is conducive to the PAAD prognostic assessment.

### 3.4 The Prognostic Value of SLC1A5 Is Validated in Multiple Cohorts

Furthermore, we tested the prognostic value of SLC1A5 in GSE62452, GSE21501, and ICGC cohorts. Intriguingly, although the GSE62452 cohort revealed that there was no significant difference in SLC1A5 expression between normal and tumor samples (Figure 2M), high SLC1A5 expression resulted in poor prognosis (HR = 1.83, P = 0.043), and achieved a predicting accuracy similar with that in TCGA cohort (AUC = 0.668) (Figures 4E,H). The expected results were also observed in GSE21501 and ICGC cohorts. SLC1A5 overexpression was still detrimental to the prognosis of patients (GSE21501: HR = 1.66, *p* = 0.042; ICGC: HR = 3.33, *p* = 0.004) (Figures 4F,G). Moreover, SLC1A5 exhibited the best predicting competency compared with other clinical characteristics in GSE21501 (AUC = 0.655) and ICGC (AUC = 0.673) cohorts (Figures 4I,J). Consequently, the prognostic value of SLC1A5 was widely confirmed.

### 3.5 High SLC1A5 Expression Hinders Antitumor Immunity and Induces the Immune-Tolerant Tumor Microenvironment

**FIGURE 5:**
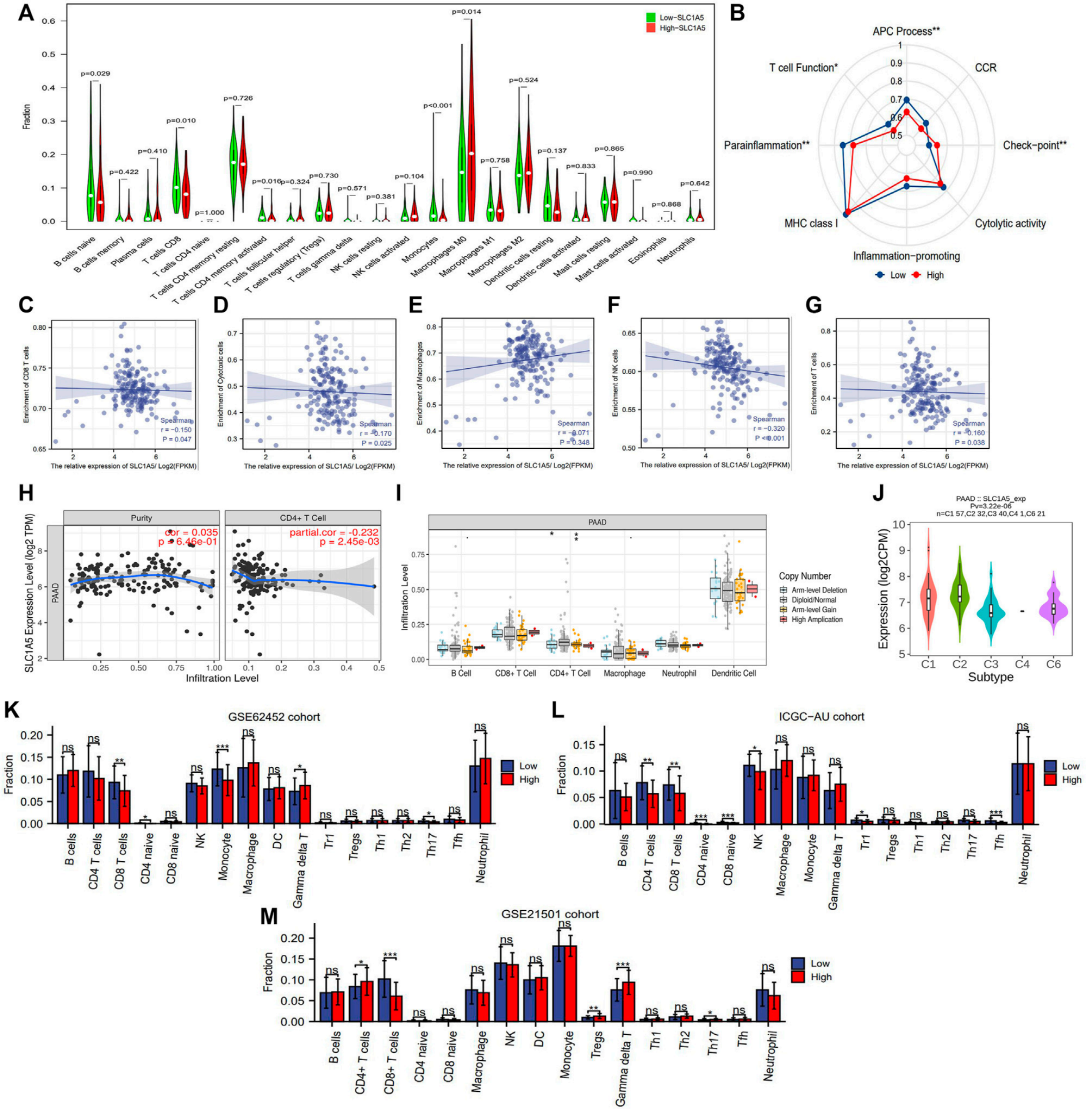
The effects of SLC1A5 on TIM. **(A)** The differences in the infiltrating levels of 22 immune cells between the high- and low-SLC1A5-expression groups (TCGA cohort). **(B)** The differences in the activities of 8 immune-related pathways between the different SLC1A5 expression groups. **(C–G)** The correlations between the SLC1A5 expression and the enrichment of five core immune cells. **(H)** The correlations between the SLC1A5 expression and the infiltrating levels of CD4^+^ T cells. **(I)** The relationship between SLC1A5 SCNA and the infiltrating levels of six crucial immune cells. **(J)** The expressive distributions of SLC1A5 in five PAAD immune subtypes. **(K–M)** The SLC1A5 expression affects the infiltrating levels of multiple immune cells in three external cohorts (GSE62452, ICGC-AU, and GSE21501). TIM, tumor immune microenvironment; PAAD, pancreatic adenocarcinoma; ICs, immune checkpoints; CD274 is also known as PD-1; NS, no statistical differences; *p < 0.05, **p < 0.01, and ***p < 0.001.

The abundance of 22 immune cells was variable in each PAAD sample (Supplementary Figure 2). High SLC1A5 expression could reduce the infiltrating levels of naïve B cells, CD8 T cells, memory-activated CD4 T cells, and monocytes. On the contrary, it could increase the immune abundance of macrophages (M0) (Figure 5A). The affected immune cells were all closely involved in the cancer immune process and progression (Table 3). As is well known, CD8^+^ T cells have abilities to kill tumor cells through perforin- and Fas/FasL-mediated apoptosis mechanisms (Wang et al., 2019). Memory CD4^+^ T cells can maintain the cytotoxic function of CD8^+^ T cells and deeply affect the clinical response of PD-1/L1 blockade therapy (Zuazo et al., 2019). Besides, the recruitment of macrophages in tumor parenchyma induces the formation of the immune-tolerant microenvironment (Nielsen and Schmid 2017). Therefore, the alterations in the infiltrating levels of these cells were detrimental to antitumor immunity.

**TABLE 3:** The effects of SLC1A5 on the infiltration levels of the immune cells.

Immune cell	PMID	Basic immune function	Trend in the high-expression group	Ultimate effect on the antitumor immune process
Naïve B cells	12969310	The naïve B cells are the source of the effector B cells and differentiated to different subtypes by chemokines	Decreased	Unfavorable
CD8 T cells	31043744	CD8 T cells exert powerful cytotoxic proficiency through perforin-granzyme and Fas-Fas ligand pathways	Decreased	Unfavorable
Memory-activated CD4 T cells	31273938	Maintain the cytotoxic function of CD8^+^ T cells	Decreased	Unfavorable
Monocytes	28052991	Monocytes induce the generation of MDSC, while the latter plays a key role in immune suppression in cancer	Decreased	Favorable
M0 macrophages	28210073	Macrophages prepare for the arrival of disseminated tumor cells and promote their invasion	Increased	Unfavorable

MDSC, myeloid-derived suppressor cells.

In addition, SLC1A5 expression was negatively correlated with immune enrichment of CD8^+^ T cells, cytotoxic cells, NK cells, CD4^+^ T cells, and total T cells (Figures 5C,D,F–H) but not with those of macrophages (Figure 5E). This indicated that SLC1A5 overexpression significantly suppressed both cellular and humoral antitumor immune processes. Besides, changes in the copy number of SLC1A5 also affected the infiltrating levels of B cells, CD4^+^ T cells, and macrophages (Figure 5I). Regarding immune-related signaling pathways, high SLC1A5 expression inhibited the activities of the antigen presentation process and T-cell function but promoted those of immune checkpoints (ICs) (Figure 5B).

It is worth mentioning that similar immune effects of SLC1A5 were also observed in three external cohorts. More specifically, high SLC1A5 expression was indicative of decreased enrichment of CD8^+^ T cells in these cohorts (GSE62452, ICGC-AU, and GSE21501) (Figures 5K–M). Collectively, SLC1A5 obviously retarded antitumor immunity by affecting the abundance of immune cells and the activities of immune-related signaling pathways.

### 3.6 SLC1A5 Is Related to the Efficacy of Dasatinib, Sunitinib, Sorafenib, and Imatinib But May Not Predict That of Radiotherapy, Chemotherapeutic Drugs, and ICIs

**FIGURE 6:**
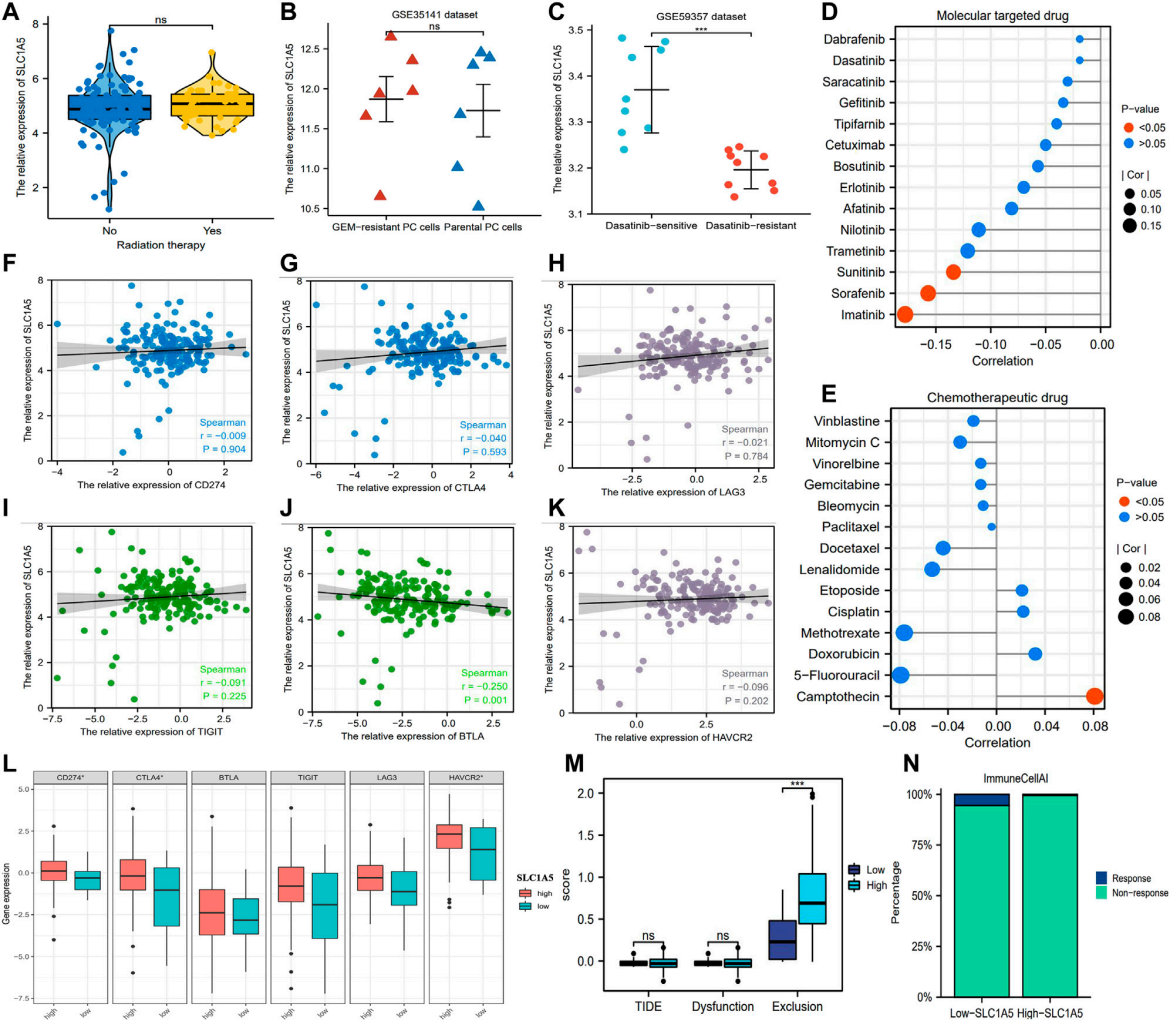
The associations of SLC1A5 with the efficacy of multiple therapeutic approaches. **(A)** The expressive difference of SLC1A5 between the radiotherapy-response and -non-response patients. **(B)** The expressive difference of SLC1A5 between gemcitabine-sensitive and -resistant cell lines. **(C)** The expressive difference of SLC1A5 between dasatinib-sensitive and -resistant cell lines. **(D)** The correlations between the SLC1A5 expression and the drug sensitivity of the MTT. **(E)** The correlations between SLC1A5 expression and the drug sensitivity of the classical chemotherapeutic agents. **(F–K)** The expressive correlations between SLC1A5 and six core ICs. **(L)** The expressive differences of six ICs between the high- and low-SLC1A5-expression groups. **(M)** The results of the TIDE immune analyses. **(N)** The predictive results of “ImmuneCellAI.” MTT, molecular target therapy; ICs, immune checkpoints; TIDE, tumor immune dysfunction and exclusion.

In the TCGA cohort, there was no obvious difference in SLC1A5 expression between radiotherapy-effective and -ineffective patients (Figure 6A). Similarly, no statistical difference in SLC1A5 expression was observed between gemcitabine-resistant and -sensitive PC cells based on the GSE35141 dataset (Figure 6B). Although SLC1A5 expression in dasatinib-sensitive PC cells was higher than that in dasatinib-resistant PC cells in the GSE59357 dataset (Figure 6C), SLC1A5 expression was not correlated with the drug sensitivity of dasatinib (Figure 6D). Among the commonly used MTT drugs, high SLC1A5 expression was accompanied with increasing drug susceptibility of sunitinib, sorafenib, and imatinib (decreased IC50) but was not associated with other ones (Figure 6D). As for chemotherapeutic agents, SLC1A5 expression has no significant linkages to their drug susceptibility but only showed a positive correlation with the IC_50_ value of camptothecin (Figure 6E).

Although there is no conclusive agreement on the biomarkers for predicting the ICI efficacy, considerable evidence has shown that patients with PD-L1 or CTLA4 overexpression possess a greater propensity for benefiting from ICI therapy (Reck et al., 2016; Zhou et al., 2020). In view of this premise, we investigated the expressive correlations between SLC1A5 and ICs. In contrast to for BTLA, CD274 (PD-L1), CTLA4, LAG3, TIGIT, and HAVCR2 were all not correlated with SLC1A5 expression (Figures 6F–K). Moreover, notable differences in the expressive levels of CD274, CTLA4, and HAVCR2 were observed between high and low SLC1A5 expressions but not in those of BTLA, TIGIT, and LAG3 (Figure 6L). Further analyses revealed that although the incidence of immune exclusion in the high-SLC1A5-expression group was notably higher than that in the low-SLC1A5-expression group, there was no difference in the TIDE score between different groups (Figure 6M). Furthermore, the proportion of patients who responded to ICIs in the low-SLC1A5-expression group (1/17, 5.88%) was higher than that in the high-SLC1A5 expression group (1/158, 0.63%) through “ImmuneCellAI” analyses (Figure 6N). Nonetheless, given that only two patients in the TCGA cohort were predicted to be responsive to ICIs, the analytical results from “ImmuneCellAI” may not have much reference significance. We speculated that the possible reason was the immune resistance derived from the pancreatic cancer (Feng et al., 2017). In brief, SLC1A5 may have a limiting competency to predict the curative efficacy of radiotherapy, chemotherapeutic drugs, MTT, and ICIs.

### 3.7 SLC1A5 Has Promoting Effects on the Proliferation, Migration, and Invasion of the Pancreatic Cancer Cells

**FIGURE 7:**
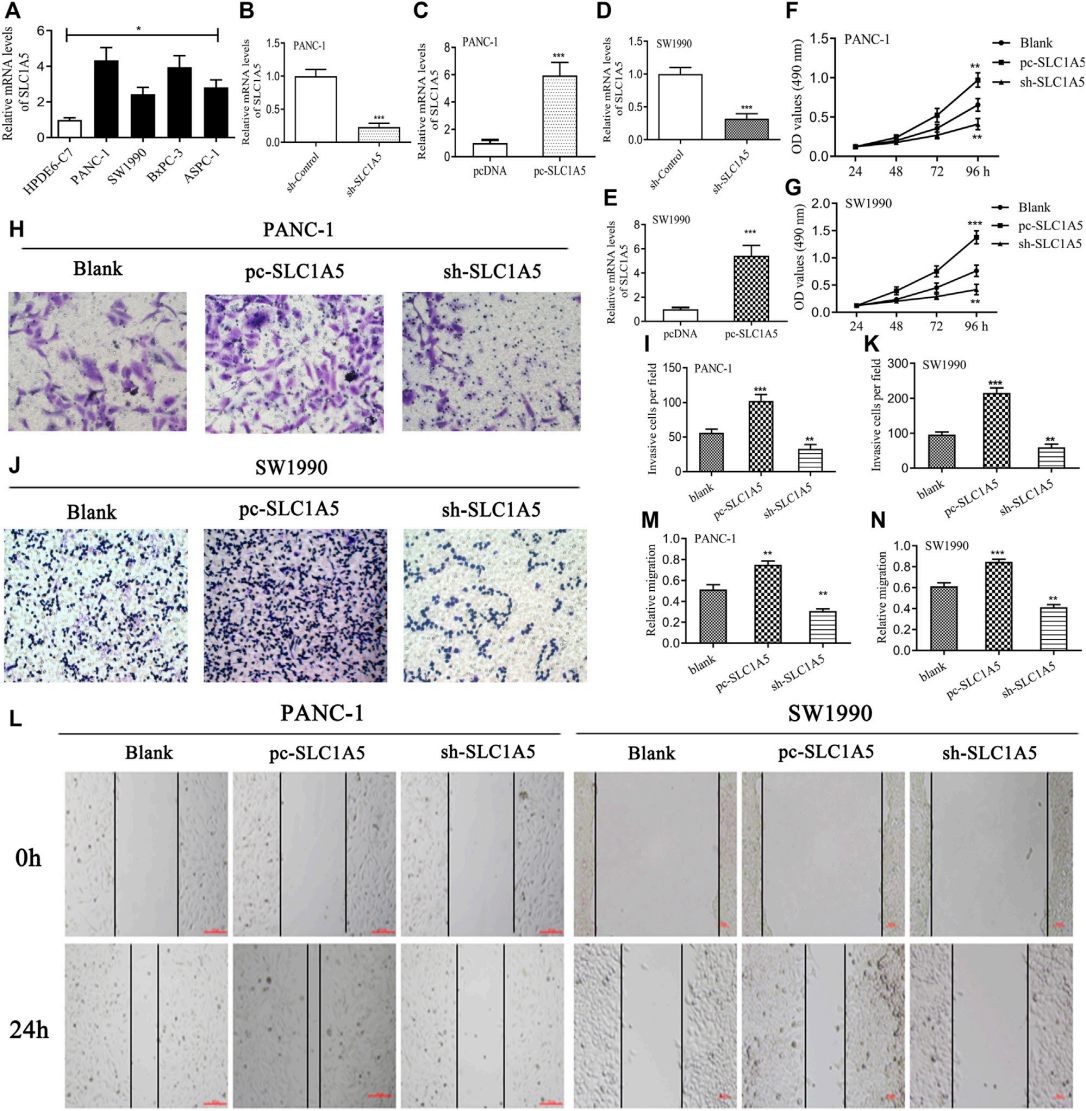
SLC1A5 has promoting effects on the proliferation, migration, and invasion of pancreatic cancer cells. **(A)** The expressive differences of SLC1A5 between the normal pancreatic duct epithelia and the PC cells. **(B–E)** Verification of the transfection efficiency in the PANC-1 and SW1990 cells. **(F,G)** Assessments of the SLC1A5 effects on the cell proliferation through the MTT assays. **(H–K)** Assessments of the SLC1A5 effects on the cell invasion through Transwell assays. **(L–N)** Assessments of the SLC1A5 effects on the cell migration through the wound-healing assays. PC, pancreatic cancer; *p < 0.05, **p < 0.01, and ***p < 0.001.

SLC1A5 expression was more significantly upregulated in the pancreatic cancer (PC) cells (PANC-1, SW1990, BxPC-3, and ASPC-1) than in the normal pancreatic duct epithelia cell (HPDE6-C7) (Figure 7A). sh-SLC1A5 and pc-SLC1A5 could effectively inhibit and increase SLC1A5 expressions (Figures 7B–E). In PANC-1 and SW1990 cells, the overexpression of SLC1A5 obviously promoted cell proliferation, whereas silencing SLC1A5 had an inhibitory effect (Figures 7F,G). Similarly, the overexpression of SLC1A5 could enhance the migration of PC cells; inversely, silencing SLC1A5 blocked that (Figures 7L–N). As for invasive ability, SLC1A5 also had a promoting effect (Figures 7H–K). Collectively, the overexpression of SLC1A5 strengthens the proliferative, migrative, and invasive abilities of PC cells.

### 3.8 SLC1A5 Activates the mTORC1 Signaling Pathway to Promote Tumor Proliferation, While It May Increase Cell Sensitivity to Ferroptosis

**FIGURE 8:**
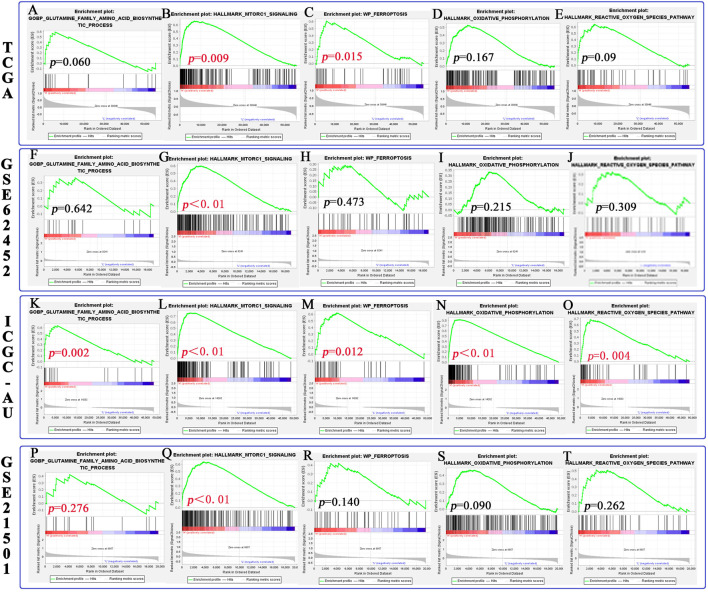
The relationship between SLC1A5 and the mTORC1 signaling pathway, glutamine metabolism, and ferroptosis process based on GSEA. **(A–E)** The differences in pathway enrichment between the high- and low-SLC1A5-expression groups in the TCGA cohort. **(F–J)** The results of GSEA in the GSE62452 cohort. **(K–O)** The results of GSEA in the ICGC-AU cohort. **(P–T)** The results of GSEA in the GSE21501 cohort. GSEA, gene set enrichment analysis.

In this study, we predicted the effects of SLC1A5 on the glutamine metabolism, mTORC1 signaling pathway, and ferroptosis process through GSEA (Table 4 and Figure 8). First, we probed into these metabolic and biological changes in the TCGA cohort (Figures 8A–E). As a glutamine transporter, SLC1A5 is capable of providing glutamine for proliferative cancer cells (Liu et al., 2018). As expected, the high SLC1A5 expression resulted in the enrichment of the “GOBP Glutamine Family Amino Acids Biosynthetic Process” (Figure 8A). In addition, the mTORC1 signaling pathway is proven to build a bridge between cell growth and nutrient metabolic processes, including proteins, lipids, and nucleotides (Ben-Sahra and Manning 2017). Meanwhile, it has been confirmed that the mTORC1 signaling pathway is closely associated with PAAD progression (Zhang et al., 2020). In light of these findings, we investigated the relationship between the pro-oncogenic abilities of SLC1A5 and the mTORC1 signaling pathway. GSEA results revealed that “Hallmark mTORC1 signaling” was significantly enriched in high-SLC1A5-expression samples (Figure 8B). From the viewpoint of ferroptosis regulation, “WP Ferroptosis” was obviously enriched in high-SLC1A5-expression samples (Figure 8C). Interestingly, the effector phase of ferroptosis, “Hallmark Oxidative Phosphorylation” and “Hallmark Reactive Oxygen Species (ROS) pathways,” did not show any enrichment (Figures 8D,E). Besides, we also investigated the expressive correlations between SLC1A5 and four ferroptosis-related markers including TFRC, SLC7A11, ACSL4, and PTGS2 based on TCGA database. As show in Supplementary Figure S3, SLC1A5 expression was positively correlated with the expressions of TFRC (R = 0.319, *p*<0.001), SLC7A11(R = 0.335, *p*<0.001) and PTGS2 (R = 0.161, *p* = 0.031), whereas it was not correlated with that of ACSL4 (R = −0.069, *p* = 0.357).

**TABLE 4:** The effects of SLC1A5 on the mTORC1 signaling pathway, glutamate metabolism, and ferroptosis process.

Gene set	Enrichment score (ES)	Normalized (NES)	Nominal *p*-value
Hallmark mTORC1 signaling	0.655	1.788	0.009
GOBP Glutamine Family Amino Acids Biosynthetic Process	0.592	1.462	0.041
WP-ferroptosis	0.607	1.696	0.015
Hallmark oxidative phosphorylation	0.526	1.353	0.167
Hallmark reactive oxygen species pathway	0.639	1.767	0.009

GSEA, gene set enrichment analysis; GO, gene ontology; BP, biological process; WP, WikiPathways.

Of note, both similar and discrepant metabolic trends could be observed in three external cohorts (Figures 8F–T and Supplementary Table 8). Similar to the TCGA cohort, the mTORC1 signaling pathway was also enriched in the samples with high SLC1A5 expression in three cohorts (Figures 8G,L,Q). Meanwhile, the effector phase of ferroptosis was not affected by the SLC1A5 expression in GSE62452 and GSE21501 cohorts (Figures 8I,G,S,T). Nevertheless, in these two cohorts, glutamine biosynthetic and ferroptosis processes were not enriched in the high-SLC1A5-expression group (Figures 8F,H,P,R), which was in contrast to the TCGA cohort. Therefore, we conducted further Western blot tests to ascertain this contradiction.

**FIGURE 9:**
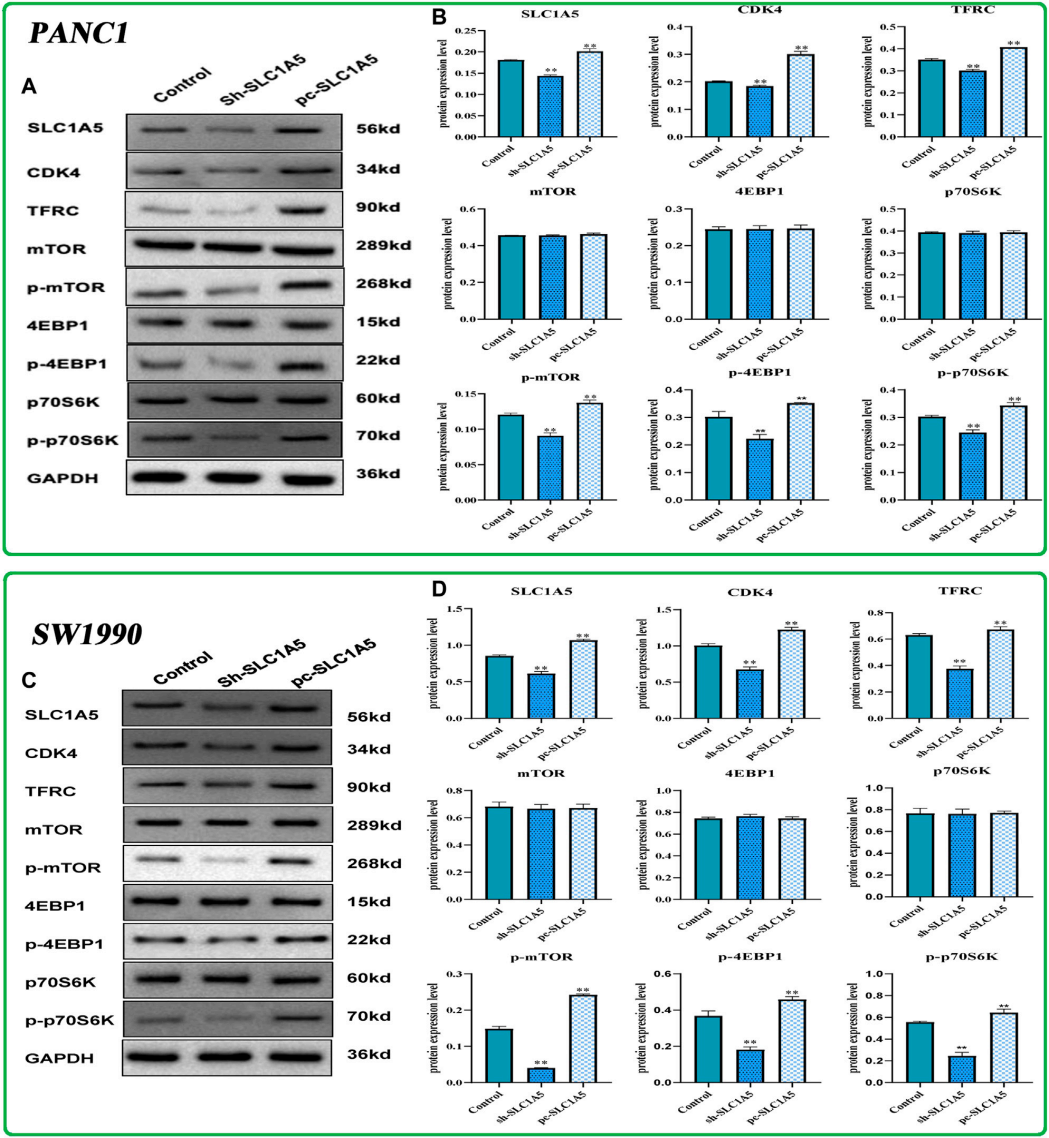
The effects of SLC1A5 on the mTORC1 signaling pathway, cell cycle, and ferroptosis based on Western blot tests. **(A,B)** The differences in the protein expression of core molecules between silencing SLC1A5 and overexpression of SLC1A5 in the PANC-1 cells. **(C,D**) The differences in the protein expression of core molecules between silencing SLC1A5 and overexpression of SLC1A5 in the SW1990 cells. *p < 0.05, **p < 0.01, and ***p < 0.001.

In PANC-1 cells, silencing SLC1A5 notably decreased the phosphorylation status of downstream effector molecules of mTORC1 pathways, including p-mTOR, p-4EBP1, and p-p70S6K, whereas the overexpression of SLC1A5 led to an opposite trend (Figures 9A,B). Meanwhile, the overexpression of SLC1A5 led to CDK4 upregulation (Figures 9A,B), indicating that SLC1A5 could facilitate proliferation of PC cells through inducing G1/S phase transition (Roskoski 2019). As acknowledged, TFRC (transferrin receptor), a hub gene responsible for iron intake, has been confirmed as a specific marker of the ferroptosis initiation (Feng et al., 2020). Besides, some researchers regard the TFRC expression level as an indicator to measure the cell sensitivity to ferroptosis (Li et al., 2019). Through Western blot tests, SLC1A5 overexpression significantly increased TFRC expression (Figures 9A,B), which suggested that SLC1A5 overexpression may enhance the sensitivity of PC cells to ferroptosis. Not surprisingly, in SW1990 cells, SLC1A5 overexpression could activate the mTORC1 pathways and upregulate the expressions of TFRC and CDK4, whereas SLC1A5 deletion conferred reverse trends (Figures 9C,D). Taking these findings into account, SLC1A5 could facilitate the proliferation, migration, and invasion of PC cells by activating the mTORC1 signaling pathway.

### 3.9 Silencing SLC1A5 Suppresses the Pancreatic Tumor Growth in a Xenograft Model

**FIGURE 10:**
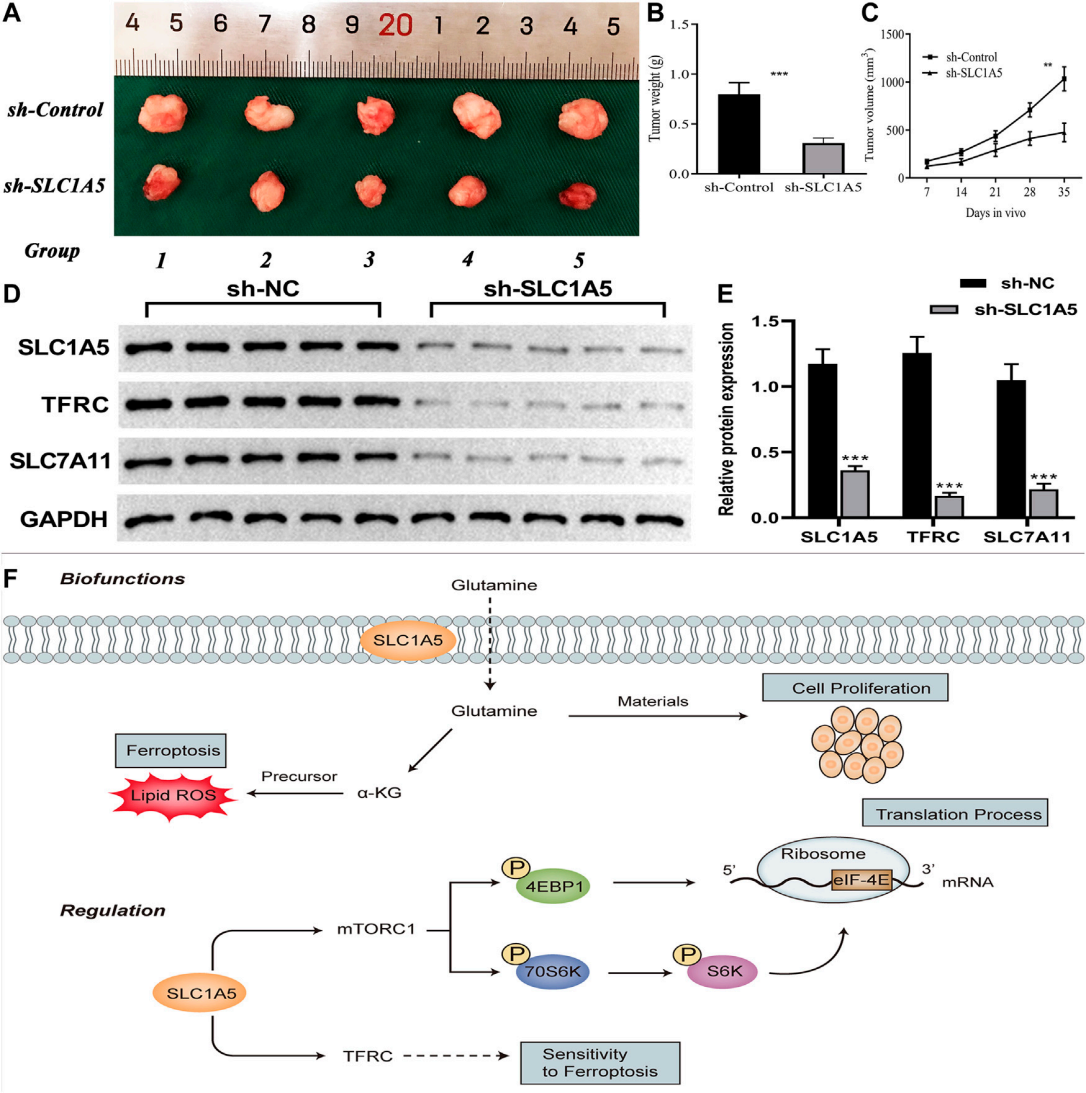
SLC1A5 presents great potentials in cancer treatment. **(A–C)** Silencing SLC1A5 suppresses pancreatic tumor growth in a xenograft model. **(D–E)** The alterations of protein expressions of SLC1A5 and four FR genes in xenograft tumors. **(F)** SLC1A5’s double-biofunctions in cancer progression (Biofunctions). SLC1A5 affects translation and ferroptosis processes through facilitating mTORC1 signaling pathway and TFRC, respectively (Regulation). FR, ferroptosis-related; *p < 0.05, **p < 0.01, and ***p < 0.001.

At the *in vivo* level, silencing SLC1A5 markedly suppressed the xenograft tumor growth (Figure 10A). The tumor weight and volume in the sh-SLC1A5 group were significantly lower than those in the negative control group (Figures 10B,C). To go a step further, we investigated the expressive alterations of SLC1A5 and ferroptosis-related genes in xenograft tumors. As shown in Figures 10D,E, the protein expressions of SLC1A5, TFRC and SLC7A11 in SLC1A5-silencing tumors were significantly lower than that in control groups. It was reasonable to believe that SLC1A5 expression was closely associated with pancreatic cancer growth. Intriguingly, the ferroptosis driver and marker, TFRC was downregulated in SLC1A5-silencing tumors, whereas the ferroptosis suppressor SLC7A11 also appeared the same tendency. One possible reason was that Fe ion and cystine that relied on TFRC and SLC7A11 transport were essential for tumor growth (Thévenod 2018; Xu et al., 2021a; Koppula et al., 2021). Briefly, targeting SLC1A5 showed promising potentials in PC treatment.

## 4 Discussion

Pancreatic adenocarcinoma is one of the most malignant tumors, the survival outcomes of which are commonly disappointing. Some limitations exist in the available therapeutic approaches, whereas ferroptosis brings a new hope for PAAD treatment. Due to the central core status in the ferroptosis process and critical function in glutamine metabolism (a double-edged sword in cancer, Figure 10F), SLC1A5 was selected as the original intention of our study. Through comprehensive bioinformatic analyses and *in vivo* and *in vitro* experimental validation, we preliminarily revealed its complex pro-oncogenic competency. SLC1A5 preferred to fulfill the proliferative demands from cancer cells rather than promoting ferroptosis to restrict PAAD progression. Moreover, SLC1A5 was of great prognostic value and had a profound impact on TIM. There is a reason to believe that our findings provide some valuable clues for mechanism exploration and clinical assessment of PAAD.

SLC1A5 plays a bidirectional role in the cancer regulation (Figure 10D). On one hand, as a ferroptotic assistant, SLC1A5 promotes intracellular glutamine accumulation and thereby enhances a-ketoglutarate synthesis (Cao and Dixon 2016). Subsequently, it in turn results in lipoperoxidation of membranes, which negatively affects cancer development by strengthening ferroptosis (Gao et al., 2015). In melanoma, miR-137 can negatively regulate ferroptosis by targeting SLC1A5 to promote cancer progression (Luo et al., 2018). On the other hand, SLC1A5 serves as a glutamine transporter that is responsible for meeting the enormous demands of glutamine for cancer cell proliferation (Liu et al., 2018). It elicits a positive function for cancer progression. For example, the overexpression of SLC1A5 facilitates tumor growth through increasing the uptake of glutamine in breast cancer (van Geldermalsen et al., 2016). So, is SLC1A5 an accomplice or an opponent in PAAD?

In this study, we confirmed that SLC1A5 could promote proliferation, migration, and invasion of PANC-1 and SW1990 cells. Meanwhile, high SLC1A5 expression was found to confer an unfavorable prognosis and to suppress the antitumor immune process. These results undoubtedly demonstrated that SLC1A5 acted as a pro-oncogenic gene in PAAD. As a glutamine transporter, SLC1A5 can notably strengthen the uptake of intracellular glutamine (Liu et al., 2018). In sum, we speculated that glutamine transported by SLC1A5 is preferentially applied to fulfill the demands of cancer cell proliferation rather than synthesizing the precursor of ferroptosis (Figure 10D). Another supportive reason is that SLC1A5 upregulation can activate the mTORC1 signaling pathway (Figure 9). Extensive research has established a central role for mTORC1 in cell proliferation (Saxton and Sabatini 2017). Activating the mTORC1 signaling pathway can increase phosphorylation of 4EBP1 and 70S6K, thereby enhancing eIF-4E stability, which is a rate-limiting factor for the translation process (Figure 10D) (Li G. et al, 2017; Kim and Guan 2019). In pancreatic cancer, miR-216 was proven to inhibit tumorigenesis by targeting the mTORC1 signaling pathway (Zhang et al., 2020). In this work, the overexpression of SLC1A5 could significantly increase the expressions of effector molecules of the mTORC1 pathway, revealing the close relationship between the cancer-promoting abilities of SLC1A5 and mTORC1 signaling pathway. Regarding ferroptosis regulation, SLC1A5 markedly upregulated the protein expression of TFRC. Given that the overexpression of TFRC can elevate the competency of cellular iron uptake that is commonly the initiation step of ferroptosis, upregulation of TFRC may imply increased cell sensitivity to ferroptosis (Feng et al., 2020). Besides, the GSEA results also revealed that although the ferroptosis process was significantly enriched in high-SLC1A5-expression samples, its effector stage “Oxidative Phosphorylation” did not present any enrichment. These findings point toward the fact that SLC1A5 could facilitate cellular sensitivity to ferroptosis but not truly trigger ferroptosis in PAAD.

Accurately predicting the prognosis of PAAD patients is not an easy task. With the application of the current eighth edition of the AJCC TNM staging system, the C-index for the 5-year OSR of PAAD patients is only 0.55 (van Roessel et al., 2018). For node-negative patients, the T stage was even not associated with survival outcomes (van Roessel et al., 2018). Moreover, a study from a Korean cancer registry found that the eighth edition N-category could not distinguish the prognostic differences of PAAD patients (Shin et al., 2019). Therefore, mono-application of TNM staging is inadequate to accurately predict the prognosis of PAAD patients. In this study, we found that the predictive performance of TNM staging was mediocre (Figures 3C, 4H–J) and only the N stage was identified as an independent prognostic factor (Figure 3G). In contrast, SLC1A5 not only increased the prognostic power of the traditional TNM model (Figure 3H) but also could distinguish the survival differences of patients with multiple clinical subgroups. There are reasons to believe that SLC1A5 contributes to the accurate prognostic assessment for PAAD patients. It was worth noting that in GSE62452 cohort, high SLC1A5 expression conferred an unfavorable prognosis, but the abnormal expression of SLC1A5 was not observed in pancreatic cancer (PC) samples compared to the normal ones (Figure 2M and Figure 4E). Currently, we have no clear and strong interpretation of this phenomenon. One possible reason is tumor heterogeneity. In addition, bioinformatic analysis may also produce some false negative results. A typical example is METTL3. Through differential expression analysis, Wang L, et al. found that METTL3 was the only m6A regulatory gene who did not differentially express in PC samples (Wang et al., 2021). Nevertheless, Xia T et al. confirmed that METTL3 was markedly upregulated in PC tissues compared to paracancerous ones through 40 clinical specimens from the first affiliated hospital of Nanjing Medical University (Xia et al., 2019).

Ferroptosis is closely related to drug resistance and immune evasion (Friedmann Angeli et al., 2019). Here, we observed that high SLC1A5 expression suppressed the infiltration levels of CD8^+^ T cells and was negatively correlated with those of NK and CD4^+^ T cells. This shows that SLC1A5 has an inhibitory effect on the antitumor immune process. Meanwhile, SLC1A5 overexpression increased the immune abundance of macrophages. Given that tumor-associated macrophages (TAMs) can mediate cancer metastasis, chemotherapeutic resistance, and immune evasion through secreting CD24, CXCL8, CCL2, and other cytokines (Petty and Yang 2017), SLC1A5 is probably involved in the formation of the immune-tolerant microenvironment. Moreover, the overexpression of SLC1A5 could suppress the activities of multiple immune-related pathways, while its alteration of the somatic copy number could affect the infiltration levels of B cells, CD4^+^ T cells, and macrophages (Figures 5B,I). These findings reiterated the pivotal roles of SLC1A5 in tumor immune regulation. In 2017, Masle-Farquhar et al. confirmed that SLC1A5-deficient mice can maintain the normal functions of B cells and humoral immunity (Masle-Farquhar et al., 2017). Targeting SLC1A5 may act as a promising strategy to trigger the antitumor immune process.

ICI therapy presents a great potential in cancer treatment. However, only a small fraction of tumor patients can benefit from this therapeutic measure. With avelumab treatment, the objective response rate of NSCLC patients was just 12% (Gulley et al., 2017). In this work, we attempted to explore the potential relationship between SLC1A5 and the ICI efficacy. High SLC1A5 expression could promote the infiltration levels of macrophages (Figure 5A). Moreover, emerging evidence has indicated that macrophage-mediated T-cell deletion restrains immunotherapy efficacy (Yu et al., 2021). In light of these findings, it seems that high SLC1A5 expression could retard the ICI efficacy. However, SLC1A5 expression was weakly correlated with six ICs (Figures 5F–L). Given that the positive PD-L1 expression is a biomarker for predicting the ICI efficacy (Conroy et al., 2019), SLC1A5 was not reliable enough to serve as a predictive indicator of the ICI response. The results of TIDE and ImmuneCellAI analyses also supported this speculation (Figures 6M,N). As for dasatinib efficacy, dasatinib-sensitive cells have a higher expression of SLC1A5 compared to dasatinib-resistant cells, indicating that patients with high SLC1A5 expression may benefit from this tyrosine kinase inhibitor (TKI) (Figure 6C). We speculated that the possible reason was that multiple TKIs, such as lapatinib and sorafenib, exert antitumor effects through inducing ferroptosis (Yu et al., 2017). Thus, the ferroptosis promoter SLC1A5 may contribute to dasatinib efficacy.

Naturally, there are some limitations that need to be noted. First, the prognostic value of SLC1A5 also needs to be validated in a real clinical cohort. Second, the carcinogenic mechanism of SLC1A5 warrants intensive study. Third, TFRC upregulation only indirectly infers the changes in sensitivity to ferroptosis but does not act as direct evidence. Fourth, by which mechanisms glutamine transported by SLC1A5 prefers to meet the demand of tumor growth rather than promoting ferroptosis is not well defined.

## 5 Conclusion

SLC1A5, a ferroptosis regulator gene, plays a dual role in cancer regulation. On one hand, it can promote the formation of a-ketoglutarate, thereby inducing ferroptosis to restrain cancer; on the other hand, it also can increase glutamine uptake to fulfill the vigorous demand for tumor proliferation. However, what role does SLC1A5 serve in PAAD? In this study, through bioinformatic analyses, the overexpression of SLC1A5 led to an unfavorable prognosis and suppressed the antitumor immune process. To go a step further, we confirmed that SLC1A5 possessed promoting effects on the proliferation, migration, and invasion of PC cells. A mice xenograft model also revealed that silencing SLC1A5 could notably suppress pancreatic tumor growth. In addition, SLC1A5 could enhance the activity of the mTORC1 signaling pathway and may increase the sensitivity of PC cells to ferroptosis through upregulating TFRC. Therefore, SLC1A5 is suspected to play as an accomplice rather than a foe in PAAD. As for therapeutic correlations, SLC1A5 may not predict the efficacy of radiotherapy, chemotherapeutic drugs, and ICIs. Our findings provide novel insights into PAAD treatment.

## Data Availability

The original contributions presented in the study are included in the article/Supplementary Material, further inquiries can be directed to the corresponding author.

## Supplementary Material

Supplementary Figure 1 The risk plots of SLC1A5 in the TCGA cohort. In total, 177 PAAD samples are divided into high- and low-SLC1A5-expression groups according to the optimal cutoff value of the SLC1A5 relative expression.

Supplementary Figure 2 The distribution of the infiltrating levels of 22 immune cells in each PAAD sample. PAAD, pancreatic adenocarcinoma.

Supplementary Figure 3 The expressive correlations between SLC1A5 and four ferroptosis-related markers based on TCGA database.

Supplementary Table 1 The primer lists.

Supplementary Table 2 Clinical characteristics of 178 PAAD patients in the TCGA cohort.

Supplementary Table 3 Clinical characteristics of 81 PAAD patients in the ICGC cohort.

Supplementary Table 4 Clinical characteristics of 65 PAAD patients in the GSE62452 cohort.

Supplementary Table 5 Clinical characteristics of 102 PAAD patients in the GSE21501 cohort.

Supplementary Table 6 The expression analyses in the TCGA cohort and the GEO datasets based on the Wilcoxon rank-sum test.

Supplementary Table 7 The results of the decision curve analysis.

Supplementary Table 8 The results of the GSEA in the GSE62452, ICGC-AU, and GSE21501 cohorts.

## Abbreviations

AJCC, American Joint Committee on Cancer; DCA, decision curve analysis; DEGs, differentially expressed genes; DSS, disease-specific survival; FRGs, ferroptosis regulatory genes; FR, ferroptosis-related; GC, gastric cancer; GEM, gemcitabine; GEO, Gene Expression Omnibus; GSEA, gene set enrichment analysis; HCC, hepatocellular carcinoma; ICIs, immune checkpoint inhibitors; ICs, immune checkpoints; ICGC, International Cancer Genome Consortium; mTORC1, mechanistic target of rapamycin complex 1; mPC, metastatic pancreatic cancer; NET, neuroendocrine tumor; NSCLC, non-small-cell lung cancer; OSR, overall survival rate; OS, overall survival; PAAD, pancreatic adenocarcinoma; PC, pancreatic cancer; PCA, pancreatic ductal adenocarcinoma; PD-1/PD-L1, programmed cell death protein/ligand; PFS, progression-free survival; PPI, protein–protein interaction; ROC, receiver operating characteristic curve; ssGSEA, single-sample gene set enrichment analysis; SPN, solid-pseudopapillary neoplasm of pancreas; SCNA, somatic copy number alteration; TCGA, The Cancer Genome Atlas; TFRC, transferrin receptor; TAMs, tumor-associated macrophages.
