# Supplementary material for: SLC1A5 Prefers to Play as an Accomplice Rather Than an Opponent in Pancreatic Adenocarcinoma
Source: Front Cell Dev Biol. 2022 Mar 28;10:800925. doi: 10.3389/fcell.2022.800925 (PMC8995533; doi:10.3389/fcell.2022.800925)
Supplement: Supplementary file 1 [file DataSheet1.zip › Supplementary Files/Supplementary table 1.docx]

Supplementary Table 1. The primer lists

| Gene | Primer | Sequence (5' -> 3') |
| --- | --- | --- |
| SLC1A5 | Ensembl number | ENSG00000105281 |
| SLC1A5 | Forward | 5′-CAGGGAGCAGGTCACAGGTC-3′ |
|  | Reverse | 5′-CAGTTTTCAGACTGCAGCAAATC-3 |
| sh-SLC1A5 | Sequence | CCGGGCCTGAGTTGATACAAGTGAACTCGAGTTCACTTGTATCAACTCAGGCTTTTTG |
| pc-SLC1A5 | Forward | 5′-GCTCTAGAATGGTGGCCGATCCTCCTCGAGACTC-3′ |
|  | Reverse | 5′-GGAATTCTTACATGACTGATTCCTTCTCAGAG-3′ |
| GAPDH | Forward | 5'‐TGCACCACCAACTGCTTAGC‐3 |
|  | Reverse | 5'‐GGCATGGACTGTGGTCATGA‐3' |
