# Supplementary material for: SLC1A5 Prefers to Play as an Accomplice Rather Than an Opponent in Pancreatic Adenocarcinoma
Source: Front Cell Dev Biol. 2022 Mar 28;10:800925. doi: 10.3389/fcell.2022.800925 (PMC8995533; doi:10.3389/fcell.2022.800925)
Supplement: Supplementary file 1 [file DataSheet1.zip › Supplementary Files/Supplementary table 2.docx]

Table 2. Clinical characteristics of 178 PAAD patients in TCGA cohort.

| Variables | Number (percentage) |
| --- | --- |
| Survival status |  |
| Alive | 86 (48.3%) |
| Dead | 92 (51.7%) |
| Age |  |
| ＜60 | 54 (30.3%) |
| ≥60 | 123 (69.1%) |
| Gender |  |
| Male | 98 (55.1%) |
| Female | 80 (44.9%) |
| Tumor Grade |  |
| G1 | 31 (17.4%) |
| G2 | 95 (53.4%) |
| G3 | 48 (26.8%) |
| G4 | 2 (1.2%) |
| Unknow | 2 (1.2%) |
| Clinical Stage |  |
| Stage I | 21 (11.8%) |
| Stage II | 146 (82.0%) |
| Stage III | 3 (1.7%) |
| Stage IV | 5 (2.8%) |
| Unknow | 3 (1.7%) |
| T stage |  |
| T1 | 7 (3.9%) |
| T2 | 24 (13.5%) |
| T3 | 142 (79.7%) |
| T4  Unknow | 3 (1.7%)  2 (1.2%) |
| M stage |  |
| M0 | 79 (44.4%) |
| M1 | 5 (2.8%) |
| Unknow | 94 (52.8%) |
| N stage |  |
| N0 | 50 (28.1%) |
| N1 | 123 (69.1%) |
| Unknow | 5 (2.8%) |

PAAD, Pancreatic adenocarcinoma; TCGA, The Cancer Genome Atlas.
