# Supplementary material for: SLC1A5 Prefers to Play as an Accomplice Rather Than an Opponent in Pancreatic Adenocarcinoma
Source: Front Cell Dev Biol. 2022 Mar 28;10:800925. doi: 10.3389/fcell.2022.800925 (PMC8995533; doi:10.3389/fcell.2022.800925)
Supplement: Supplementary file 1 [file DataSheet1.zip › Supplementary Files/Supplementary table 3.docx]

Supplementary Table 3. Clinical characteristics of 81 PAAD patients in ICGC cohort.

| Variables | Number (percentage) |
| --- | --- |
| Survival status |  |
| Alive | 32 (39.5%) |
| Dead | 49 (60.5) |
| Age |  |
| ＜65 | 33 (40.7%) |
| ≥65 | 47 (58.1%) |
| Unknown | 1 (1.2%) |
| Gender |  |
| Male | 40 (49.4%) |
| Female | 41 (50.6%) |
| Survival time (day) (49 dead samples) |  |
| ≤365 | 25 (51.0%) |
| 365＜Time≤730 | 17 (34.7%) |
| 730＜Time≤1095 | 3 (6.1%) |
| ＜1095 | 4 (8.2%) |
| Follow up (day) (32 alive samples) |  |
| ≤365 | 11 (34.4%) |
| 365＜Time≤730 | 9 (28.1%) |
| 730＜Time≤1095 | 11(34.4%) |
| ＜1095 | 1 (3.0%) |
| TNM-system, clinical stage, and tumor grade | Unknown |

PAAD, Pancreatic adenocarcinoma; ICGC, International Cancer Genome Consortium.
