# Supplementary material for: SLC1A5 Prefers to Play as an Accomplice Rather Than an Opponent in Pancreatic Adenocarcinoma
Source: Front Cell Dev Biol. 2022 Mar 28;10:800925. doi: 10.3389/fcell.2022.800925 (PMC8995533; doi:10.3389/fcell.2022.800925)
Supplement: Supplementary file 1 [file DataSheet1.zip › Supplementary Files/Supplementary table 4.docx]

Supplementary Table 4. Clinical characteristics of 65 PAAD patients in GSE62452 cohort.

| Variables | Number (percentage) |
| --- | --- |
| Survival status |  |
| Alive | 16 (24.6%) |
| Dead | 49 (75.4%) |
| Tumor Grade |  |
| G1 | 2 (3.2%) |
| G2 | 32 (49.2%) |
| G3 | 29 (44.6%) |
| G4 | 1 (1.5%) |
| Unknown | 1(1.5%) |
| Clinical stage |  |
| Stage I | 4 (6.2%) |
| Stage II | 45 (69.2%) |
| Stage III | 10 (15.4%) |
| Stage IV | 6 (9.2%) |
| Age, gender, and TNM stages | Unknown |

PAAD, Pancreatic adenocarcinoma.
