# Supplementary material for: SLC1A5 Prefers to Play as an Accomplice Rather Than an Opponent in Pancreatic Adenocarcinoma
Source: Front Cell Dev Biol. 2022 Mar 28;10:800925. doi: 10.3389/fcell.2022.800925 (PMC8995533; doi:10.3389/fcell.2022.800925)
Supplement: Supplementary file 1 [file DataSheet1.zip › Supplementary Files/Supplementary table 5.docx]

Supplementary Table 5. Clinical characteristics of 102 PAAD patients in GSE21501 cohort.

| Variables | Number (percentage) |
| --- | --- |
| Survival status |  |
| Alive | 36 (35.3%) |
| Dead | 66 (64.7%) |
| T stage |  |
| T 1 | 2 (1.9%) |
| T 2 | 16 (15.7%) |
| T 3 | 79 (77.5%) |
| T 4 | 1 (1.0%) |
| Unknown | 4 (3.9%) |
| N stage |  |
| N 0 | 28 (27.5) |
| N 1 | 73 (71.5) |
| Unknown | 1 (1.0%) |
| Age, gender, tumor grade, clinical stage, and M stage | Unknown |

PAAD, Pancreatic adenocarcinoma.
