# Supplementary figures and images for: SLC1A5 Prefers to Play as an Accomplice Rather Than an Opponent in Pancreatic Adenocarcinoma
Source: Front Cell Dev Biol. 2022 Mar 28;10:800925. doi: 10.3389/fcell.2022.800925 (PMC8995533; doi:10.3389/fcell.2022.800925)

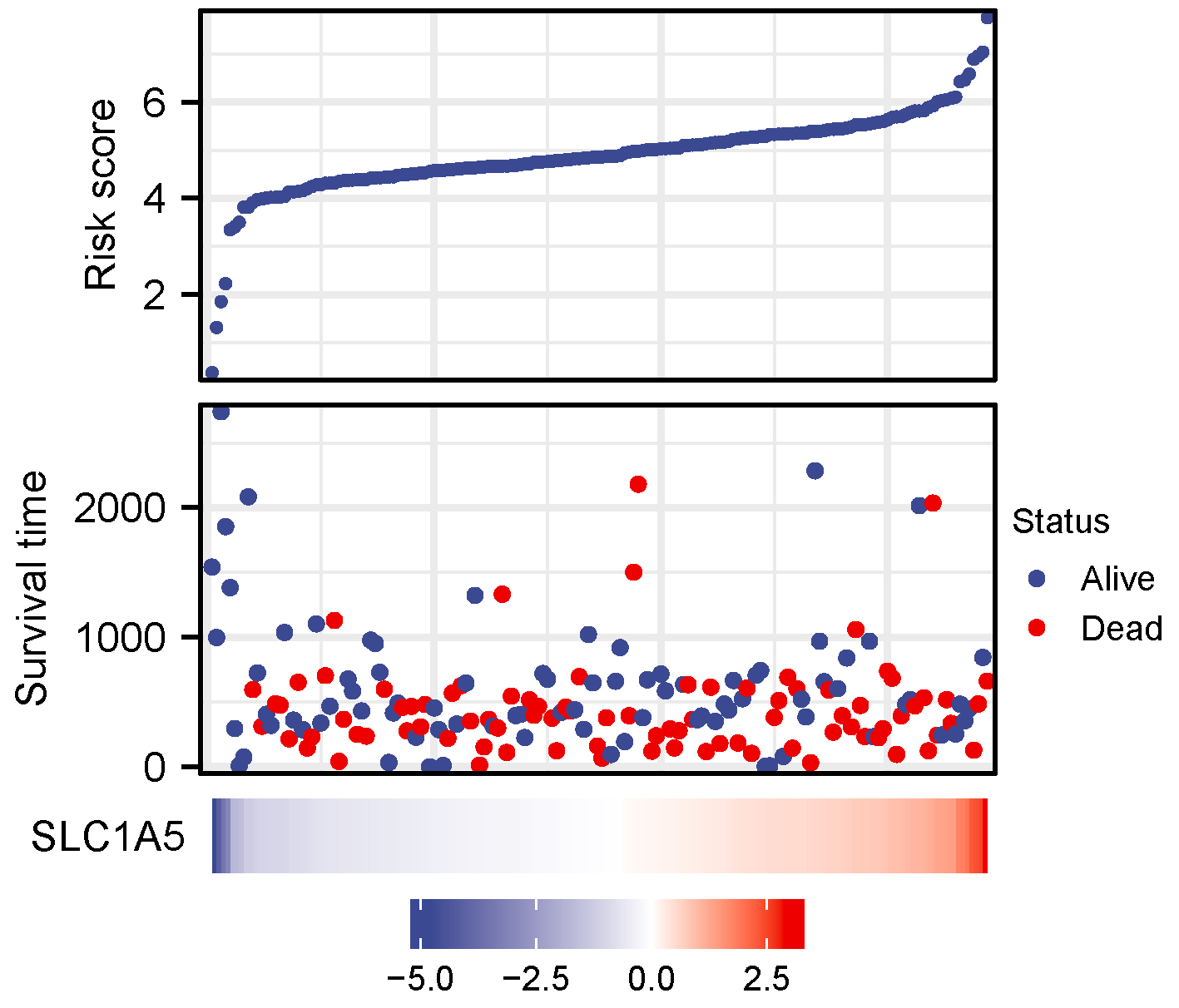

Supplement: Supplementary file 1 [file DataSheet1.zip › Supplementary Files/Supplementary figure 1.jpg]

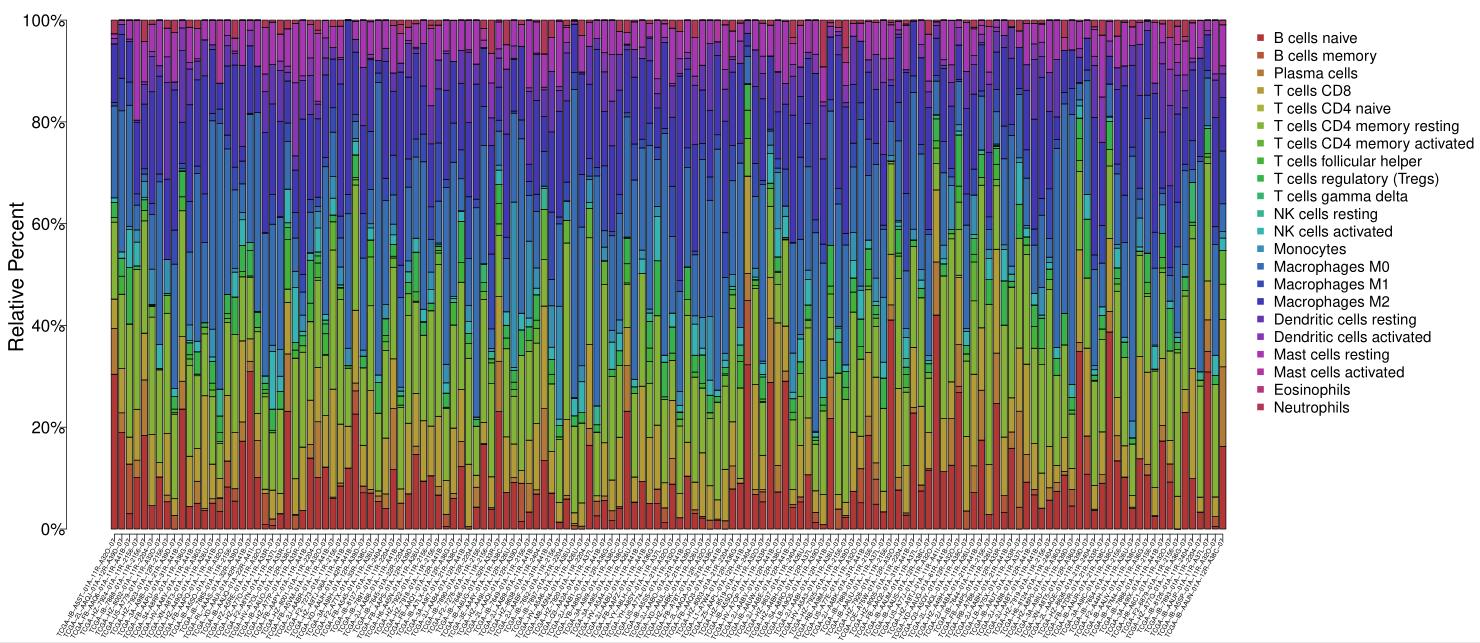

Supplement: Supplementary file 1 [file DataSheet1.zip › Supplementary Files/Supplementary figure 2.jpg]

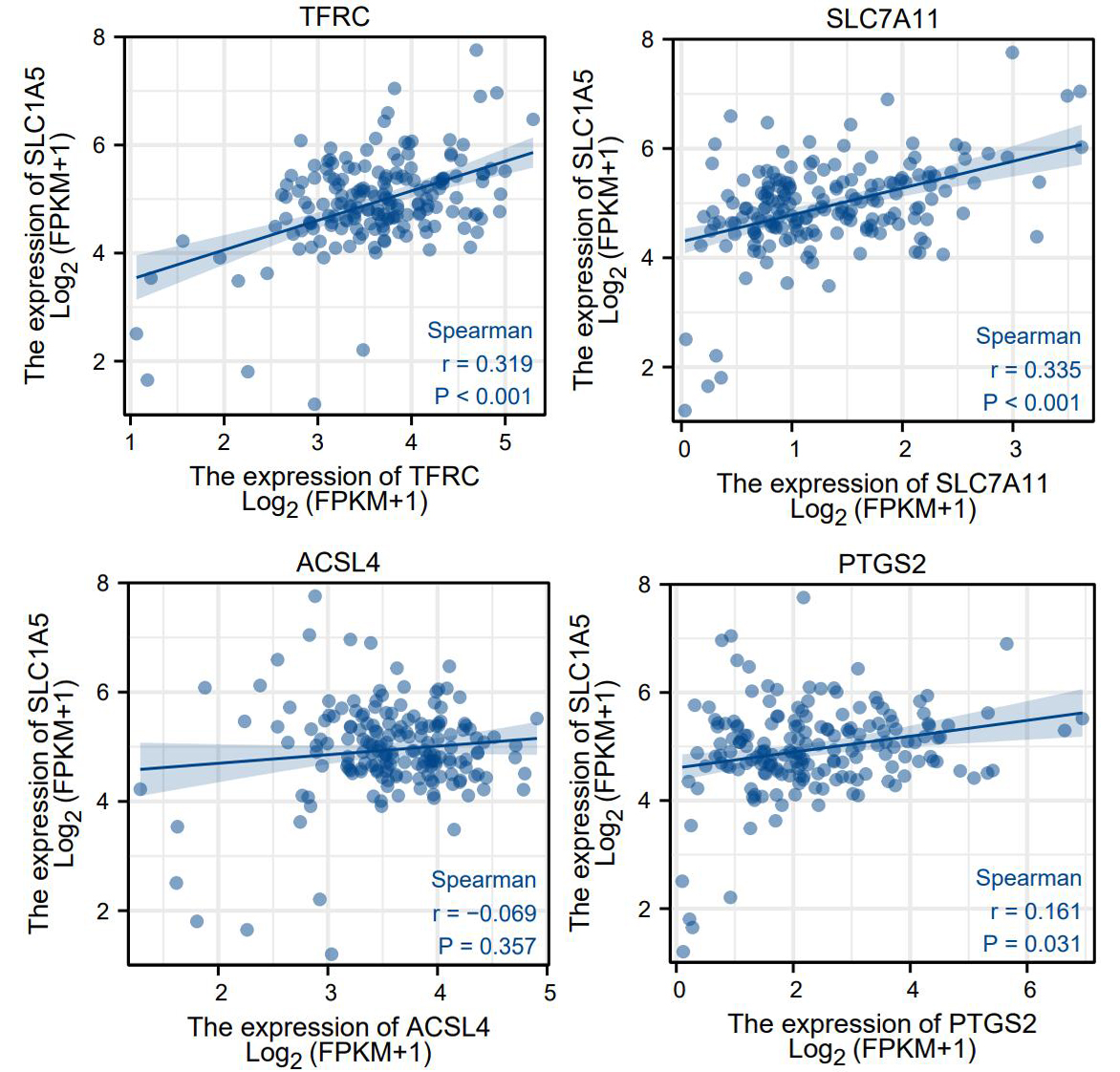

Supplement: Supplementary file 1 [file DataSheet1.zip › Supplementary Files/Supplementary figure 3.jpg]
